# Serine Phosphorylation of HIV-1 Vpu and Its Binding to Tetherin Regulates Interaction with Clathrin Adaptors

**DOI:** 10.1371/journal.ppat.1005141

**Published:** 2015-08-28

**Authors:** Tonya Kueck, Toshana L. Foster, Julia Weinelt, Jonathan C. Sumner, Suzanne Pickering, Stuart J. D. Neil

**Affiliations:** Department of Infectious Disease, King’s College London School of Life Sciences and Medicine, Guy’s Hospital, London, United Kingdom; University of Wisconsin, UNITED STATES

## Abstract

HIV-1 Vpu prevents incorporation of tetherin (BST2/ CD317) into budding virions and targets it for ESCRT-dependent endosomal degradation via a clathrin-dependent process. This requires a variant acidic dileucine-sorting motif (ExxxLV) in Vpu. Structural studies demonstrate that recombinant Vpu/tetherin fusions can form a ternary complex with the clathrin adaptor AP-1. However, open questions still exist about Vpu’s mechanism of action. Particularly, whether endosomal degradation and the recruitment of the E3 ubiquitin ligase SCF^βTRCP1/2^ to a conserved phosphorylated binding site, DSGNES, are required for antagonism. Re-evaluation of the phenotype of Vpu phosphorylation mutants and naturally occurring allelic variants reveals that the requirement for the Vpu phosphoserine motif in tetherin antagonism is dissociable from SCF^βTRCP1/2^ and ESCRT-dependent tetherin degradation. Vpu phospho-mutants phenocopy ExxxLV mutants, and can be rescued by direct clathrin interaction in the absence of SCF^βTRCP1/2^ recruitment. Moreover, we demonstrate physical interaction between Vpu and AP-1 or AP-2 in cells. This requires Vpu/tetherin transmembrane domain interactions as well as the ExxxLV motif. Importantly, it also requires the Vpu phosphoserine motif and adjacent acidic residues. Taken together these data explain the discordance between the role of SCF^βTRCP1/2^ and Vpu phosphorylation in tetherin antagonism, and indicate that phosphorylation of Vpu in Vpu/tetherin complexes regulates promiscuous recruitment of adaptors, implicating clathrin-dependent sorting as an essential first step in tetherin antagonism.

## Author Summary

Counteraction of tetherin, a host antiviral protein that blocks viral release from infected cells, is an essential attribute of HIV-1 and its related viruses. The HIV-1 accessory protein Vpu binds to tetherin, preventing its incorporation into viral particles, and targets it for ubiquitin-dependent degradation. This involves mis-trafficking of tetherin by a Vpu-dependent mechanism through the engagement of clathrin adaptor proteins. Although structural evidence exists for Vpu and tetherin interacting with clathrin adaptor 1 (AP-1), evidence that it is required for Vpu-mediated tetherin counteraction is still lacking. Tetherin degradation by Vpu also requires an E3 ubiquitin ligase, SCF^βTRCP1/2^ that binds to phosphorylated serine residues in the Vpu cytoplasmic tail. Again, discrepancies exist about the importance of this interaction in tetherin’s counteraction. Here we show that Vpu phosphorylation, in combination with its physical interaction with tetherin, regulates interaction with both AP-1 and the other major cellular clathrin adaptor, AP-2. These interactions can be decoupled from SCF^βTRCP1/2^ recruitment, thus indicating clathrin-dependent mis-trafficking as a critical step in tetherin antagonism by Vpu. Additionally, the ability to interact both with AP-1 and AP-2 in a tetherin-dependent manner indicates a redundancy in host cofactors used by Vpu that explains disparate previous observations of its mechanism of action.

## Introduction

Counteraction of the antiviral membrane protein tetherin (BST2/ CD317) is an essential attribute of primate lentiviruses, and is mediated by either the Vpu or Nef accessory proteins, or occasionally the viral envelope glycoprotein (reviewed in [1]). In their absence, tetherin restricts the release of virions assembling at the cell surface [2–6]. By virtue of its N-terminal transmembrane (TM) domain and C-terminal GPI anchor, partitioning of tetherin dimers into budding virions allows them to simultaneously span host and viral membranes resulting in accumulation of cross-linked virions on the plasma membrane (PM) [7,8]. In addition to physically limiting virion release, tetherin’s activity sensitizes infected cells to antibody-dependent cellular cytotoxicity [9–12], targets virions for endosomal degradation, and in the case of great ape tetherins, can directly induce the activation of proinflammatory NF-**κ**B signaling [13–16].

Tetherin recycles to the PM via the trans-Golgi network (TGN) [17]. This requires a dual tyrosine-based sorting signal (YDYCRV in humans), which can interact with the clathrin adaptor AP-1. Lentiviral countermeasures physically interact with tetherin, often in a highly species-specific manner [1]. Through their action, tetherin incorporation into virions is blocked, and this is associated with its reduced cell surface levels. In the case of HIV-1 Vpu, a small membrane phospho-protein, physical interaction is mediated by the TM domains themselves [18–20]. HIV-1 Vpu targets human tetherin into an ESCRT-dependent endosomal degradation pathway [21,22]. This is an ubiquitin driven process and requires a highly conserved DSGNES motif in the Vpu cytoplasmic tail [23–25]. Phosphorylation of the serine residues (S52/53 and S56/57 in subtype B depending on the isolate) by casein kinase II (CKII) [26,27] recruits the β-TrCP1/2 subunits of a Skp1-Cullin1-F-Box (SCF) E3 ubiquitin ligase [28] that mediates direct ubiquitination of various residues in the tetherin cytoplasmic tail including an STS motif [29]. However, there is still debate as to whether the recruitment of the SCF^βTRCP1/2^ to the DSGNES motif in Vpu is required for counteraction of physical retention of virions by tetherin (hereafter also termed antagonism) as well as its final endosomal degradation. Much of this discrepancy may be attributable to whether assays are performed in virally infected cells or those transiently transfected with Vpu, tetherin or both [30]. While ESCRT-I appears to be dispensable in infected cells [21], evidence that the ESCRT-0 component HRS is required for tetherin antagonism suggests targeting to endosomal degradation plays a role [22]. Furthermore, mutations of the Vpu serine residues (so called 2/6 mutations) have intermediate phenotypes in tetherin antagonism suggesting degradation does not fully explain Vpu function[24,25,31]. Moreover this defect in antagonism is not recapitulated by siRNA depletion of β-TrCP1/2 [32]. Indeed evidence that the DSGNES motif might have a dual function in tetherin trafficking has been proposed [33]. This is consistent with our recent study of Vpu variation in patients where we found that naturally occurring variants in the NE of the DSGNES imparted tetherin-specific defects to Vpu without blocking its other SCF-dependent activity, dislocation of CD4 from the endoplasmic reticulum [34].

Vpu has been shown to block newly synthesized and/or recycling tetherin from trafficking to the cell surface [33,35]. This requires a variant of an acidic dileucine motif, ExxxLV, in the second alpha helix of the cytoplasmic tail of most HIV-1 group M clade Vpu [36]. Acidic dileucine sorting signals bind to the σ subunits of the major cellular clathrin adaptors AP-1 (trafficking from TGN to endosomes and *vice versa*) and AP-2 (clathrin-dependent endocytosis from the PM) (reviewed in [37]). In keeping with this, Vpu-mediated tetherin antagonism is entirely clathrin-dependent [36,38]. Mutation of the ExxxLV motif does not block Vpu/tetherin interactions, but reduces the efficiency of counteraction and inhibits degradation [36]. In particular ExxxLV is essential for counteraction of tetherin in CD4+ T cells upon interferon upregulation, and mutant phenotypes are exacerbated when tetherin lacks the YDYCRV motif [36]. A recent structural and biochemical study has demonstrated that the ExxxLV motif can bind canonically to the σ subunit of AP-1, whereas the YXXθ motif of tetherin can bind to the μ subunit of AP-1 [39]. In fusions of Vpu and tetherin cytoplasmic tails both motifs can occupy their respective binding sites simultaneously [39]. Some density in the structure also indicated other contacts between Vpu and AP-1μ, and together implied a mechanism whereby the formation of this ternary complex would modulate AP-1-dependent trafficking of tetherin to endosomes. However, whilst the localization of Vpu to the TGN suggested AP-1 as the major target, siRNA-mediated knockdown of AP-1 or expression in AP-1 -/- murine fibroblasts did not inhibit Vpu function [36]. Neither has physical interaction between AP-1 and the wild-type Vpu protein been demonstrated in living cells. Expression of tetherin fused at its N-terminus to the second helix of Vpu is excluded from budding virions at the PM in an ExxxLV-dependent manner [18]. Added to this, tetherin can be expressed as two isoforms, one of which lacks the YDYCRV motif and can be antagonized by Vpu to a certain extent without cell surface downregulation [13,40]. Likewise, Vpu has only a modest effect on tetherin endocytosis [25,35], and AP-2 knockdown also has little impact on antagonism, contrasting sharply with SIV Nef and HIV-2 envelopes [38,41,42].

AP1 binding to a non-canonical acidic dileucine motif in CI-M6PR has been associated with upstream serine phosphorylation by CKII previously [43]. Thus we hypothesized that the DSGNES in Vpu might regulate clathrin adaptor interaction independently of SCF recruitment. Here we provide evidence that this is indeed the case.

## Results

### Vpu does not require ESCRT-I, HRS or β-TrCP to counteract tetherin in HIV-1 infected cells

**Fig 1:**
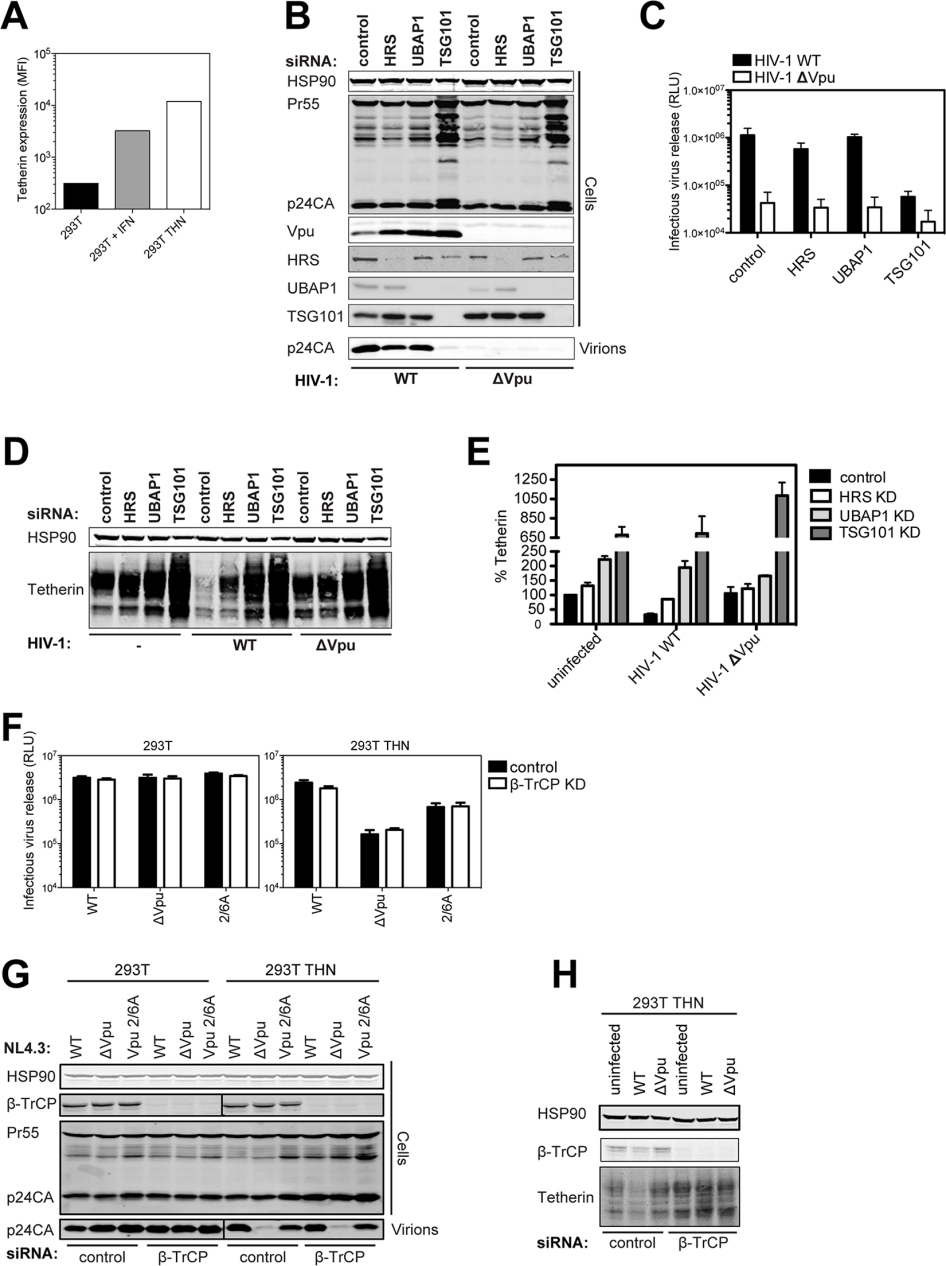
Vpu does not require ESCRT-I, HRS or βTRCP to counteract tetherin in HIV-1 infected cells. (A) The graph indicates the median fluorescence intensity of tetherin surface expression for 293T cells, 293T cells treated with 1000 U/ml universal type-I interferon for 24h or the same cells stably expressing tetherin. (B) 293T tetherin cells were transfected twice over a 48 hour period with siRNA oligonucleotide directed against HRS, UBAP1, TSG101 or non targeting control. Cells were then infected with NL4.3 HIV-1 WT or HIV-1 ΔVpu at an MOI of 0.8. Cell lysates and sucrose purified viral supernatants were subjected to SDS-PAGE and analyzed by Western blotting for HSP90, HIV-1 p24CA and Vpu, and analyzed by LiCor quantitative imager. (C) Infectivity of viral supernatants from (B) was assayed on HeLa-TZMbl reporter cells. Infectious virus release was plotted as β-galactosidase activity in relative light units (RLU). Error bars represent the standard deviation of three independent experiments. (D) Cells were treated as in (B), but infected with an MOI of 2. Cell lysates were subjected to SDS-PAGE and analyzed by Western blotting for HSP90 and tetherin, and analyzed by LiCor quantitative imager. (E) Percent of tetherin in cells transfected with HRS or non-targeting siRNA oligonucleotides and infected with NL4.3 HIV-1 WT or HIV-1 ΔVpu. Error bars represent the standard deviation of three independent experiments. (F-H) 293T or 293T tetherin cells were transfected as in (B) with siRNA oligonucleotide directed against β-TrCP1 and 2 or non-targeting control. Cells were infected with VSV-G pseudotyped NL4.3 HIV-1 WT, ΔVpu or Vpu 2/6A mutant at an MOI of 0.8 and processed as in (C) and (D). (H) Cells were treated as in (F) but infected at an MOI of 2. Cell lysates were subjected to SDS-PAGE and analyzed by Western blotting for HSP90 and tetherin.

The importance of the SCF^βTRCP1/2^ E3 ligase and the ultimate degradation of tetherin to the counteraction of its physical antiviral activity by Vpu has been controversial. Since the discrepant studies were mostly performed under conditions of transient transfection of tetherin, provirus or both, and which have been shown previously to lead to artifactual effects on tetherin degradation [30], we re-examined these issues in HIV-1 infected 293T cells stably expressing surface tetherin at levels similar to those induced by type 1 interferon (Fig 1A). We have previously shown that an endosomal sorting-specific subunit of ESCRT-I, UBAP1, is essential for tetherin’s degradation but not for antagonism [21,36]. Despite efficient levels of knockdown, similarly efficient siRNA knockdowns of HRS (ESCRT-0) or UBAP1 had only minor effects on one-round yield of wild-type HIV-1 (HIV-1 wt) from 293T tetherin cells at an MOI of 0.8 (Fig 1B and 1C). As expected, knockdown of the core ESCRT-I subunit TSG101 destablized UBAP1 [44] and blocked all virion release because of its essential late-domain function [45], and all siRNA treatments also stabilized Vpu expression (Fig 1B). In keeping with this, cells infected at an MOI of 2, to ensure at least 90% infection, demonstrated that Vpu-induced degradation was blocked by all siRNA knockdowns (Fig 1D and 1E). These data therefore indicate that in infected cells expressing physiological levels of Vpu from an integrated HIV-1 provirus, the core ESCRT pathway and HRS are essential for Vpu-mediated tetherin degradation, but dispensable for counteraction of tetherin’s physical antiviral activity.

A previous study indicated that HRS interacted with Vpu in immuno-precipitates [22]. We confirmed this in transfected cells using myc-tagged HRS, and found that HRS truncations that removed its double-ubiquitin interaction motif (DUIM) inhibited this interaction (S1A and S1B Fig). Furthermore, point mutations in the DUIM that abolish ubiquitin-interaction (A266Q/ A228Q) [46], not putative ubiquitin-binding mutants in the VHS domain, completely abolished HRS/Vpu interactions in co-IPs (S1C Fig). Whilst formally possible that the DUIM is a direct binding site for Vpu, these data likely suggest that Vpu interactions with HRS are mediated indirectly through ubiquitination either of cargo, or associated factors in the degradation pathway.

We next similarly re-evaluated the effect of simultaneously knocking down β-TrCP1 and 2 on Vpu-mediated tetherin-degradation and tetherin-counteraction in infected cells. Again despite efficient knockdown, we saw little effect of this treatment on HIV-1 WT release (Fig 1F–1H). Of note, there was no evidence that β-TrCP1/2 knockdown reduced wild-type release to that of a viral mutant lacking the phosphorylated serines at positions 52 and 56 that are essential for β-TrCP1/2 recruitment (HIV-1 Vpu 2/6A). This was in contrast to a complete reversal of Vpu-mediated tetherin degradation by β-TrCP1/2 siRNAs in cells infected at an MOI of 2 (Fig 1H). Therefore whilst tetherin degradation by Vpu requires the SCF^βTRCP1/2^ complex, under conditions when it is sufficiently depleted to block this, there is no effect on Vpu-mediated tetherin antagonism.

### Phosphorylation-defective Vpu phenocopies trafficking mutants

**Fig 2:**
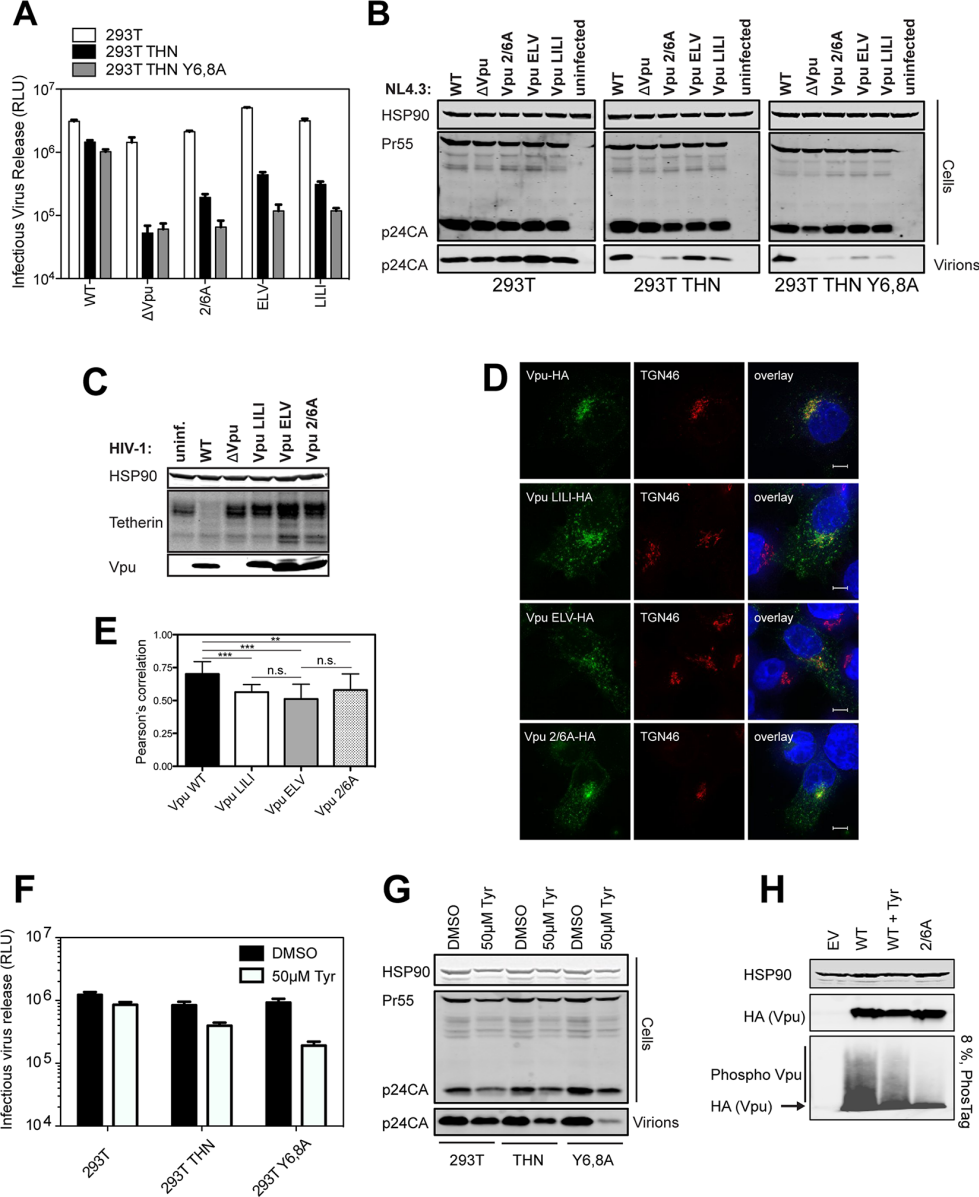
Phosphorylation-defective Vpu phenocopies trafficking mutants. (A-B) 293T, 293T tetherin or Y6,8A tetherin cells were infected with VSV-G pseudotyped NL4.3 WT or mutant virus at an MOI of 0.8. (A) 48 hours post infection viral supernatants were assayed for infectivity using HeLa-TZMbl reporter cells as in Fig 1. Error bars represent the standard deviation of three independent experiments. (B) Cell lysates and sucrose purified viral supernatants were subjected to SDS-PAGE and analyzed by Western blotting as in Fig 1. (C) 293T tetherin cells were infected with NL4.3 HIV-1 WT, ΔVpu, Vpu LILI, Vpu ELV or Vpu 2/6A mutants at an MOI of 2. 48 hours post infection cell lysates were subjected to SDS-PAGE and analyzed by Western blotting for HSP90 and tetherin, and analyzed by LiCor quantitative imager. (D) 293T tetherin expressing cells were transfected with 50 ng of pCR3.1 Vpu-HA or indicated mutants. 16 hours post transfection cells were fixed and stained for HA (green) and the TGN marker TGN46 (red) and examined by widefield fluorescent microscopy. Panels are of representative examples. Bars = 10 μm. (E) Z stacks were taken of all cells (n = 15), images were deconvolved using the AutoQuant X3 software and Pearson’s correlations were calculated for all Z stacks using ImageJ. Results were analyzed by unpaired 2-tailed t-test—*** P = 10–5 or lower. (F) 293T, 293T tetherin or Y6,8A tetherin cells were infected with VSV-G pseudotyped NL4.3 WT at an MOI of 0.8. 6 hours post infection DMSO or 50 μM Tyrphostin was added to the medium. 48 hours post infection supernatants were assayed as in (A). (G) Cell lysates and sucrose purified viral supernatants were processed as in (B). (H) 293T tetherin cells were transfected with 2 μg pCR3.1 Vpu-HA or 2/6 Vpu-HA and treated with DMSO or 50 μM Tyrphostin for 24 h. Cell lysates were electrophoresed as before, or on a 8%, 50 μM Phos-tag gel to separate the phosphorylated species.

Since the phospho-mutant of Vpu, Vpu 2/6A, has been shown to be partially defective for tetherin antagonism [23–25], we revisited whether this impairment could be uncoupled from the ubiquitin ligase. We recently showed that mutants of clade B Vpu lacking a conserved ExxxLV sorting signal (Vpu ELV) were also partially defective for tetherin antagonism because they could not traffic tetherin/Vpu complexes for endosomal degradation [36]. Notably, ELV mutant Vpu loses all residual activity against tetherin lacking the dual-tyrosine recycling motif, and a recent study demonstrated that the tetherin and Vpu cytoplasmic tails can assemble into a ternary complex with clathrin adaptor AP-1 [39]. In addition, hints in the structure suggested that residues 42 and 43 of the first helix of the cytoplasmic tail make a non-canonical contact with AP-1**μ**. We found similar Vpu mutants with tetherin-defective phenotypes in our patient cohort [34], and mutation of conserved L_41_I_42_/L_45_I_46_ in the first alpha helix to alanines in the NL4.3 provirus led to a profound defect in tetherin antagonism and degradation without preventing interaction (S2A–S2E Fig). Since the DSGNES motif is located in an acidic patch between helix 1 and the ExxxLV site, we hypothesized that Vpu phosphomutants may also be similarly defective for mis-trafficking tetherin. In one round virus infection assays in 293T/tetherin cells, LI/LI, ELV and 2/6A mutants all had similarly defective phenotypes for tetherin antagonism (Fig 2A and 2B). Interestingly, like the ELV mutant [36,39], both LI/LI and 2/6A mutants lost all their residual activity in cells expressing tetherin Y6,8A whereas release of the wild-type virus was only slightly affected. Moreover, as expected, all mutants were defective for tetherin degradation (Fig 2C). Examination of the localization of the three mutants in transfected HeLa cells revealed that, unlike the wild-type, 2/6A and LILI localized prominently to peripheral endosomal structures as well as the TGN (Fig 2D). This was similar to the localization expected for the ELV mutant [36], and quantification of coincidence with TGN46 revealed that all three mutants had a significantly reduced localization to the TGN consistent with a trafficking defect (Fig 2E). Importantly there was no significant additive effect of combined 2/6 and ELV mutations in full-length virus release from either the 293T/tetherin cells or primary CD4+ T cells (S3A–S3C Fig). Also these data could be recapitulated using a highly active primary Vpu (Vpu 2_87) isolate from our previous patient study [34] (S4A–S4D Fig). Treatment of 293T tetherin cells infected with wild-type HIV-1 with a CKII inhibitor, Tyrphostin, to mimic the 2/6A mutation showed a reduction of virus release only in the presence of tetherin, or more prominently, the Y6,8A mutant (Fig 2F and 2G). Western blot analysis of cell lysates transfected with HA-tagged Vpu expression vectors and run on an 8% PhosTag gel showed that in the presence of Tyrphostin, the smear of phosphorylated Vpu was reduced indicating inhibition of Vpu phosphorylation (Fig 2H). Together, these data therefore suggested that the defective tetherin antagonism of Vpu 2/6A may be due to phosphorylation-regulated trafficking of Vpu rather than ubiquitin ligase recruitment and degradation.

### Functional rescue of Vpu phospho- and trafficking mutants by direct interaction with clathrin

**Fig 3:**
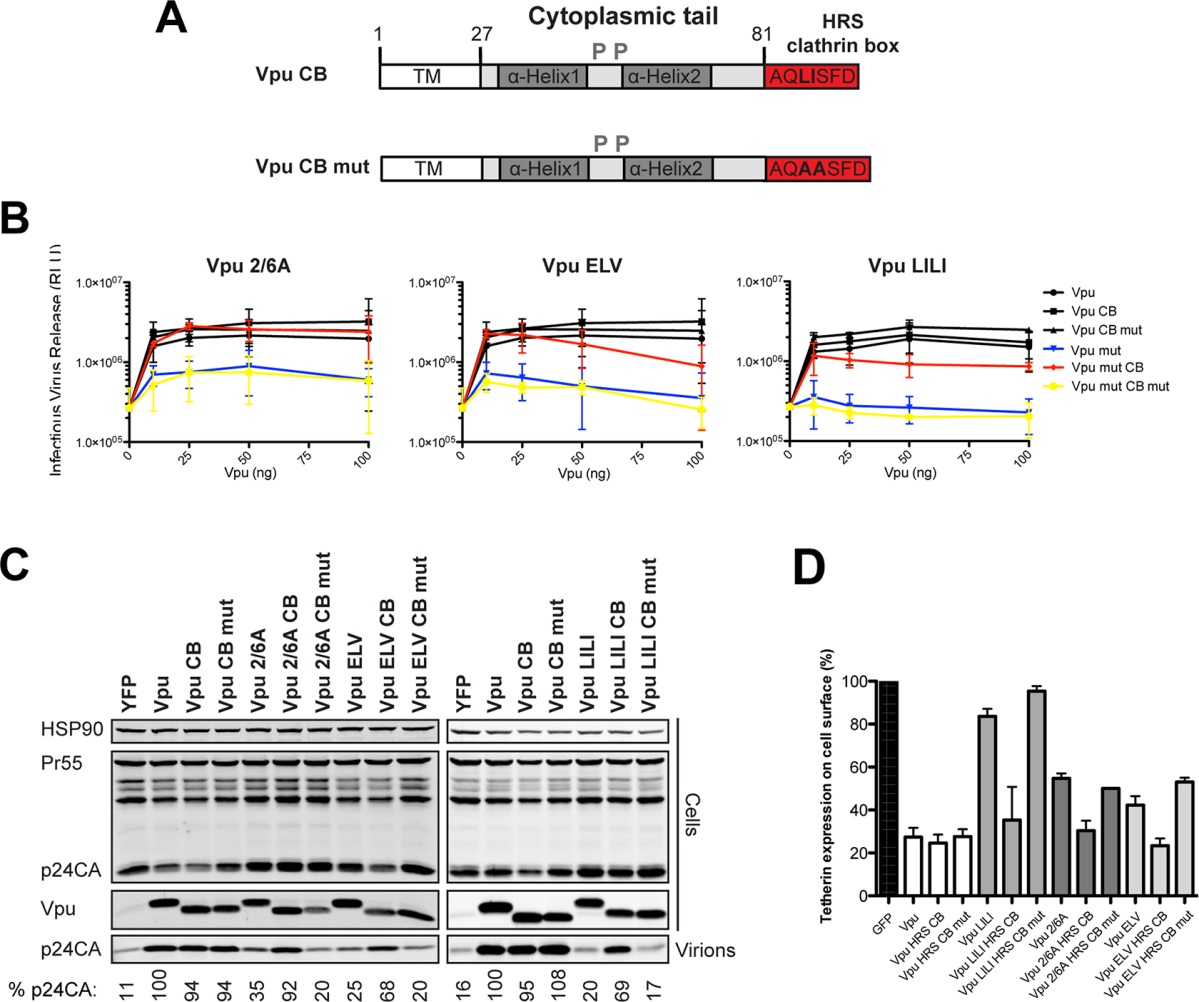
Functional rescue of Vpu phospho- and trafficking mutants by direct interaction with clathrin. (A) Schematic representation of Vpu CB chimera constructs. (B) 293T tetherin cells were transfected with NL4.3 ΔVpu proviral plasmid in combination with YFP expression vector and pCR3.1 Vpu, pCR3.1 Vpu CB or Vpu CB mut or Vpu mutants thereof. 48 hours post transfection infectivity of viral supernatants was determined on HeLa-TZMbl cells as in Fig 1. Error bars represent standard deviation of three independent experiments. (C) Cell lysates and pelleted supernatant virions from (B) were harvested and subjected to SDS-PAGE and analyzed by Western blotting for HIV-1 p24CA, Vpu and HSP90, and analyzed by LiCor quantitative imager. (D) HeLa-TZMbl cells were co-transfected with pCR3.1 Vpu or indicated mutant and a GFP expression vector. Cell-surface tetherin levels were analyzed 48 hours post transfection by flow cytometry in the GFP positive cells. The percentages of tetherin surface expression levels are calculated from median fluorescence intensities.

**Fig 4:**
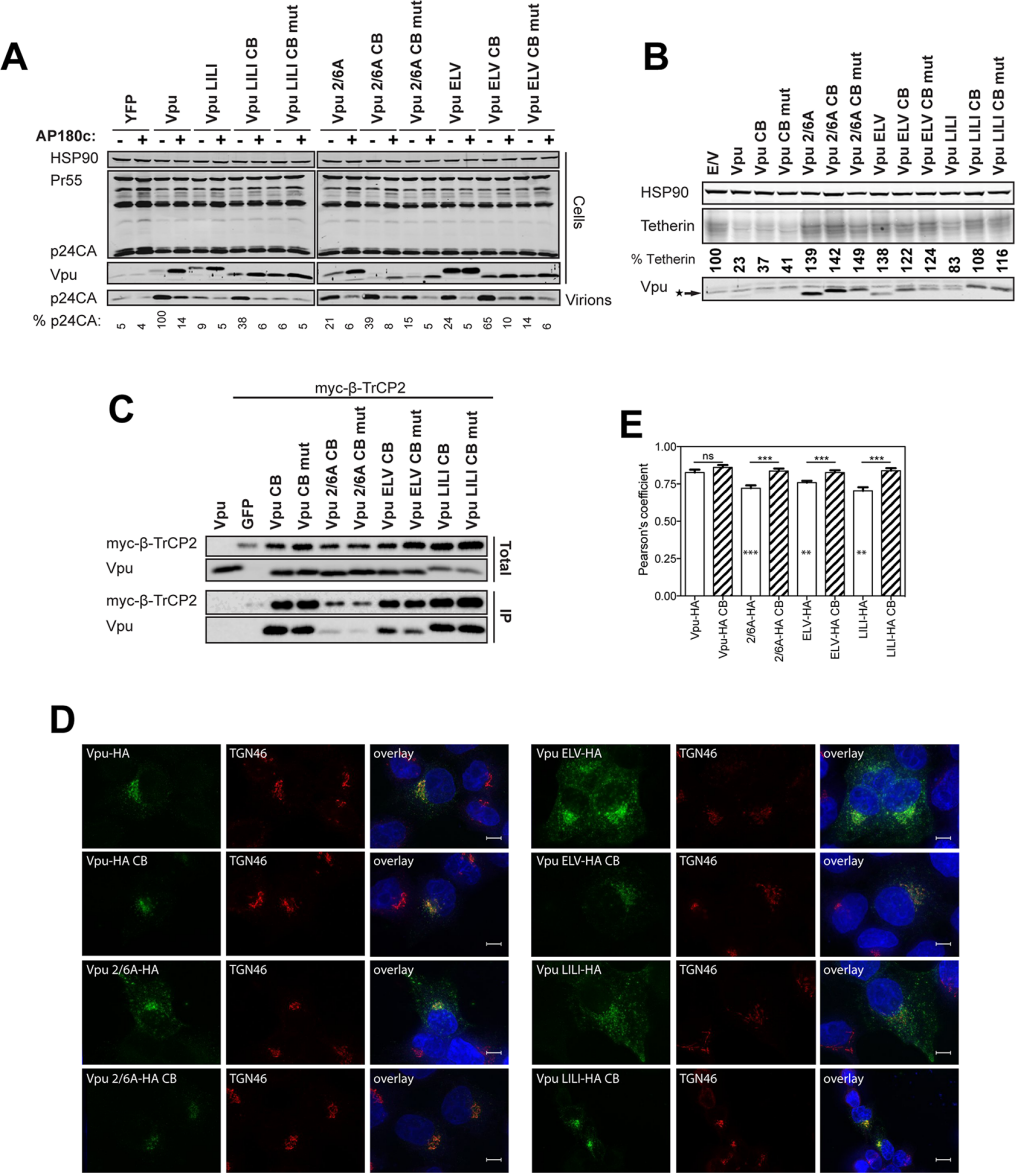
Clathrin binding rescues Vpu localization without restoring β-TrCP binding or tetherin degradation. (A) 293T tetherin expressing cells were co-transfected with NL4.3 ΔVpu proviral plasmid and YFP, pCR3.1 Vpu, Vpu CB, Vpu CB mut expression vector or indicated mutant in combination with AP180c. 48 hours post transfection cell lysates and pelleted supernatant virions were harvested and subjected to SDS-PAGE and analyzed by Western blotting for HIV-1 p24CA, Vpu and HSP90, and analyzed by LiCor quantitative imager. (B) 293T tetherin cells were transfected with pCR3.1 Vpu or indicated mutant. 48 hours post infection cell lysates were subjected to SDS-PAGE and analyzed by Western blotting for HSP90, tetherin and Vpu, and analyzed by LiCor quantitative imager. (C) 293T cells were transfected with pCR3.1 Vpu or indicated mutant in combination with a pCR3.1 myc-β-TrCP2 expression vector. 48 hours post transfection cells were lysed and immunoprecipitated with anti-myc antibody, using PFA (0.05% w/v) as a cross-linking agent. Total cell lysates and precipitates were subjected to SDS-PAGE and analyzed by Western blotting for myc-β-TrCP2 and Vpu-HA, and analyzed by ImageQuant. (D-E) Hela cells were transfected with 100 ng of pCR3.1 Vpu-HA or Vpu-HA CB or indicated mutants and processed as in Fig 2. Vpu-HA (green); TGN marker TGN46 (red). (G) Bars = 10 μm. (E) Asterisks inside the bars represent significant localization differences between the mutant and WT Vpu, those above between mutant and mutant clathrin box fusion.

The current model for Vpu function is that it prevents tetherin trafficking to the PM from the TGN and sorts it into a clathrin-dependent endosomal trafficking pathway [1,47]. If our above hypothesis was the case, we reasoned that bypassing clathrin adaptors and linking Vpu directly to clathrin itself could functionally rescue all ELV, LI/LI and 2/6A mutants. To do this we appended the AQLISFD clathrin box (CB) from HRS or a mutated sequence, AQAASFD, lacking the leucine and isoleucines essential for clathrin interaction, to the C-termini of Vpu and the respective mutants (Fig 3A). Transient transfection of increasing doses of Vpu into 293T tetherin cells effectively rescued Vpu-defective HIV-1 viral release, and neither the clathrin box nor its mutant impaired wild-type Vpu function (Fig 3B and 3C). Remarkably, however, Vpu 2/6A, Vpu ELV or Vpu LI/LI function was almost fully restored by fusion of the clathrin box, whereas grafting the mutated sequence had no effect. All Vpu chimeras were well expressed, although as shown in Fig 3C, the apparent molecular weight of Vpu and its chimeras in SDS-PAGE did not reflect amino acid length. Similar results were obtained for a heterologous clathrin box (RNLLDLL) derived from GGA2 (available on request). The clathrin box also fully restored downregulation of tetherin from the surface of transiently transfected HeLa-TZMbl cells to all the mutants (Figs 3D and S5A–S5D). To show that this rescue of function was clathrin-dependent, we depleted clathrin membrane binding with the C-terminal fragment of the neuronal clathrin-adaptor AP180 (AP180c). As expected, rescue of wild-type Vpu-dependent virus release was inhibited by AP180c whereas residual viral release in the presence of tetherin was not [36]. In all cases, the same held true for clathrin box fusions (Fig 4A). Thus, direct linkage to the clathrin machinery was sufficient to rescue both Vpu 2/6A and the trafficking mutants. Moreover, in cells stably expressing the Vpu chimeras, no reduction of tetherin steady state levels was observed upon CB fusion to any of the chimeras (Fig 4B), nor was β-TrCP interaction restored to the 2/6A mutant fusion (Fig 4C), indicating this was independent of SCF and ESCRT function. Wild-type subcellular localization was restored to all mutants; 2/6A, ELV and LI/LI localization was significantly restored to TGN-associated compartments upon CB fusion (Fig 4D and 4E).

**Fig 5:**
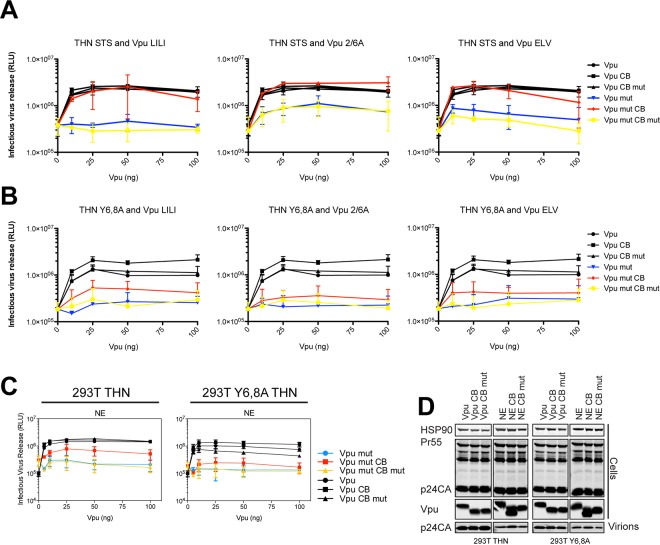
Clathrin box rescue of Vpu mutants is dependent on tetherin’s Y6,8 sorting signal. (A-B) 293T tetherin STS or 293T tetherin Y6,8A cells were transfected with NL4.3 ΔVpu proviral plasmid in combination with YFP expression vector and increasing concentrations of pCR3.1 Vpu or indicated mutant. 48 hours post transfection infectivity of viral supernatants was determined on HeLa-TZMbl cells as in Fig 1. (C) 293T tetherin or 293T tetherin Y6,8A cells were transfected as in (A) with pCR3.1 Vpu or the N54H,E55G (NE) mutant. 48 hours post transfection infectivity of viral supernatants was determined on HeLa-TZMbl cells as in Fig 1. Error bars represent standard deviation of three independent experiments. (D) Cell lysates and pelleted supernatant virions from (A) were harvested and subjected to SDS-PAGE and analyzed by Western blotting for HIV-1 p24CA, Vpu and HSP90, and analyzed by LiCor quantitative imager.

To further characterize these Vpu chimeras, we next examined whether they were functional against tetherin bearing tyrosine (trafficking) and serine/threonine (the proposed SCF^βTRCP^ ubiquitination site [29]) mutations in the cytoplasmic tail. In the case of 293T tetherin-STS-AAA cells, the Vpu CB chimeras behaved as they did against the wild-type protein, effectively fully rescuing the 2/6A, LILI or ELV lesion (Fig 5A). Importantly, stable expression of an STS mutant tetherin had no detectable effect on the efficiency of counteraction by wild-type Vpu, and the CB addition had no effect, indicating that there is no reduction in Vpu antagonism when tetherin lacks the residues proposed to be important for ubiquitination. However, in the case of 293T tetherin Y6,8A cells, whilst Vpu wild-type and CB fusions remained active, the mutant chimeras remained completely defective (Fig 5B). These data imply that unlike the ExxxLV motif, the clathrin box addition is not dominant over the tetherin tyrosine-based sorting motif. This therefore suggests that tetherin sorting into clathrin-rich domains in the recycling compartment is essential for clathrin box chimera rescue, which then anchors the Vpu/tetherin complex. Subsequent endosomal trafficking, and importantly, any requirement for serine/threonine ubiquitination are downstream of this event. It also further reinforces the notion that the primary lesion in tetherin antagonism of the 2/6A mutant, like ELV and LI/LI, is at the level of clathrin-dependent sorting, not ubiquitin ligase recruitment.

Finally we examined mutations within the DSGNES motif itself. The consensus for a β-TrCP-binding site is DSGxxS, yet the N55/E56 in group M Vpu is almost universally conserved. We found rare mutations (N55H/E56G) in patients that displayed impaired tetherin antagonism despite retaining β-TrCP interaction [34]. Similarly, examination of a Vpu N55H/E56G mutation in the context of the NL4.3 Vpu revealed defects in tetherin counteraction in 293T tetherin cells (S3 and S4 Figs), which again could be rescued by a clathrin box fusion unless tetherin itself contained tyrosine mutations (Fig 5C and 5D). Together with the above data, these observations suggest that structural constraints or flexibility within the phosphoserine motif may underlie the reason why the 2/6A mutant is defective for tetherin mis-trafficking.

### Vpu interacts with clathrin adaptors AP-1 and AP-2 in tetherin-expressing cells

Our previous characterization of the ExxxLV motif and the data presented herein indicate that clathrin-dependent sorting of Vpu/tetherin complexes is an essential step in tetherin antagonism, prior to ubiquitin-dependent degradation. The demonstration that the ExxxLV motif of Vpu and the YDYCRV site in tetherin can form a ternary complex with AP-1 [39] is consistent with the cell biological observations that Vpu primarily blocks tetherin recycling and transit to the PM rather than stimulating its endocytosis [33,35]. However, demonstration that Vpu can interact with AP-1 in cells is lacking, and neither siRNA depletion of AP-1, nor deletion of γ-adaptin in murine fibroblasts, affects tetherin antagonism [36]. Clathrin adaptor interactions with their cargoes can sometimes (but not universally) be detected in yeast 2 or 3-hybrid assays or with recombinant proteins, but the relative weakness of their affinities often precludes direct demonstration of their interactions *in vivo* by conventional immunoprecipitations. To examine Vpu interaction with AP-1, we initially employed a proximity-based biotin ligase assay (S6A Fig). A consenus clade B Vpu or indicated mutant (note the phosphomutant S53,57A is labeled S3/7A), was fused to a myc-tagged E coli biotin ligase BirA-R113G, which itself does not compromise Vpu activity (S6B Fig). These constructs were then transfected into 293T or 293T tetherin cells. 6 hours after transfection the cells were incubated with free-biotin overnight in the presence of concanamycin A to block any tetherin degradation by the wild-type Vpu protein. Cell lysates were precipitated with streptavidin beads, and recovered proteins analyzed by Western blotting. Such treatment will lead to promiscuous biotinylation of proteins in close proximity with Vpu, potentially allowing us to detect interacting factors with weak affinities. As shown in S6C Fig, addition of biotin led to an accumulation of biotinylated proteins in cell lysates, including a strong band that is auto-biotinylation Vpu-BirA fusion itself. Importantly, β-TrCP was detected for all mutants tested in both 293T and 293T tetherin cells except the 2/6A mutant. Interestingly AP-1 γ-adaptin was detected only in streptavidin precipitates from 293T tetherin cells transfected with wild-type Vpu-BirA fusion, and not cells lacking tetherin expression. Furthermore, in 293T tetherin cells both ELV and LI/LI mutants failed to biotinylate AP-1. Interestingly, this was observed for the 2/6A mutant and also a Vpu A14L/W22A mutant that lacks tetherin binding. Thus, proximity-based tagging suggested Vpu does indeed interact with AP-1 in living cells. This appears to be dependent on tetherin binding and requires both the predicted AP-1σ binding site in Vpu, ExxxLV, and the non-canonical AP-1μ contact proposed to imparted by LI/LI. Furthermore, the lack of the 2/6A mutant to biotinylate AP-1γ suggests that Vpu phosphorylation is required to promote interaction, consistent with its cellular phenotype.

**Fig 6:**
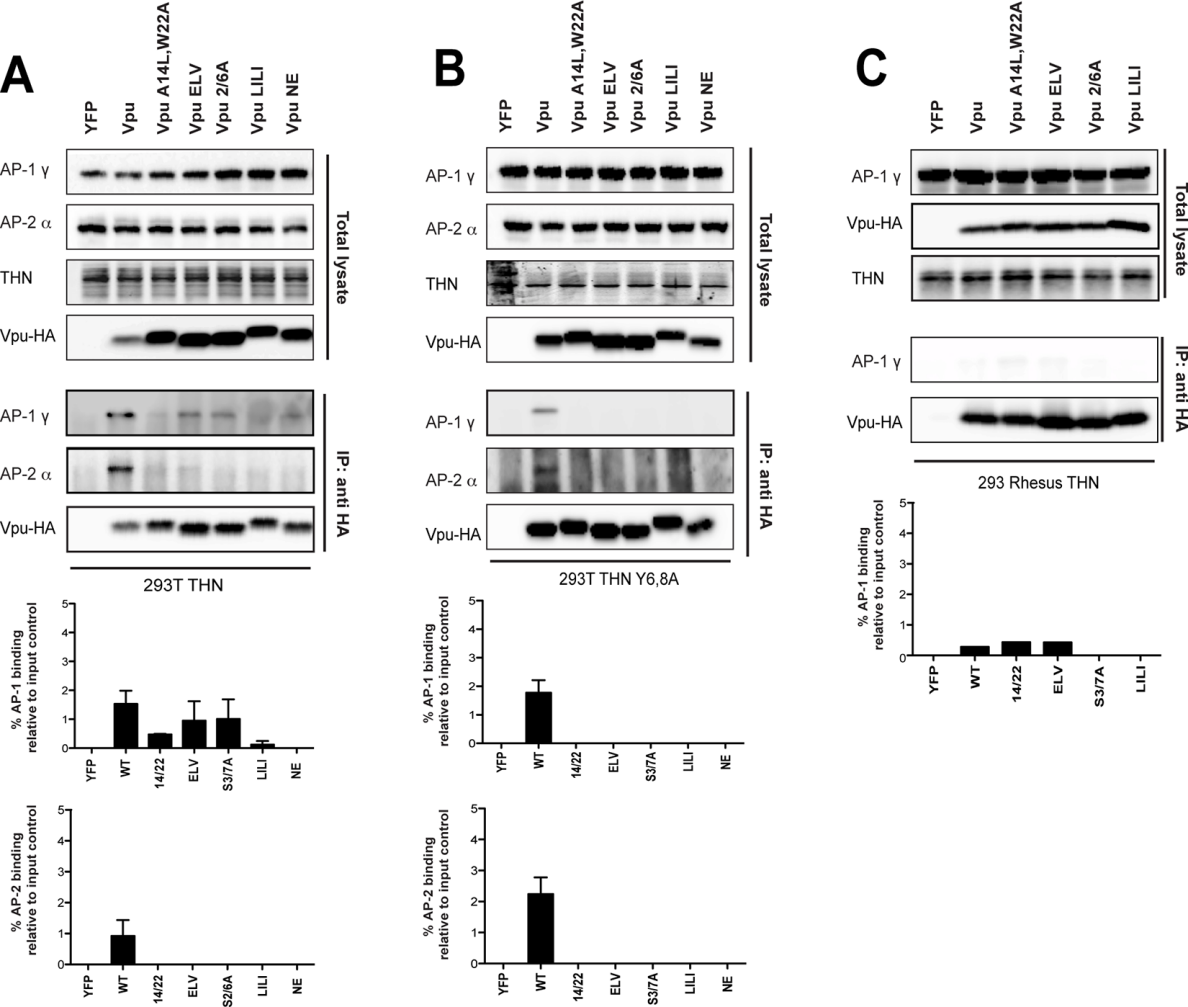
Vpu interacts with clathrin adaptors AP-1 and AP-2 in tetherin-expressing cells. (A-C) 293T tetherin (A), 293T tetherin Y6,8A (B) or 293 rhesus tetherin (C) cells were transfected with pCR3.1 Vpu-HA, Vpu A14L/W22A-HA, Vpu ELV-HA, Vpu 2/6A-HA, Vpu LILI-HA or Vpu NE-HA mutants. 48 h post transfection, cells were lysed and cross-linked using PFA (0.05% w/v) and immunoprecipitated with anti-HA antibody. Total cell lysates and precipitates were subjected to SDS-PAGE and analyzed by Western blotting for Vpu-HA, tetherin, AP-1γ or AP-2α. Panels are of representative experiments. Histograms represent western blot quantification of the relative AP-1 or AP-2 binding normalized to input control. Error bars represent the standard deviation of three independent experiments.

Whilst this data is strongly suggestive, it does not rule out that conformational changes in the mutants position the BirA in a context where AP-1 cannot be biotinylated. To strengthen these observations, we performed cross-linking immunoprecipitations in 293T tetherin cells transfected with HA-tagged Vpu or all of the above Vpu mutants. This revealed that AP-1γ could be detected in immunoprecipitates of Vpu-HA (Fig 6A). This was not detected for the A14L/W22A mutant, again indicating a requirement for tetherin interaction. A reduced amount of AP-1γ was detected in the 2/6A and ELV mutant immunoprecipitates, and this varied between replicates (see histogram below blot). Since tetherin’s YDYCRV motif also binds to AP-1 (Jia et al., 2014), we repeated the immunoprecipitations in 293T tetherin Y6,8A cells (Fig 6B). Whilst AP-1 precipitation was preserved for the wild-type protein, this effectively removed all detectable AP-1 interactions with any of the mutants, including the NE mutation between the two serines, indicating the reduced detection was due to tetherin/AP-1 interactions. To confirm these data, we also performed the same precipitations in 293T cells expressing a rhesus macaque tetherin to which HIV-1 Vpu cannot bind (Fig 6C), or parental 293T cells (S7A Fig) and found that no AP-1 could be detected under any conditions. These data also held true for the patient isolate Vpu 2_87 (S7B Fig). Therefore, these data demonstrate for the first time that Vpu does interact with AP-1 *in vivo*. Tetherin/Vpu TM-domain interactions are essential for this interaction, as are the predicted AP-1 binding sites in Vpu. Moreover, the lack of interaction of the 2/6A mutant indicates that phosphorylation of Vpu upstream of the ExxxLV regulates AP-1 interaction, and these data correlate well will the clathrin dependency presented in Fig 4.

The ExxxLV motif has the potential to bind to other clathrin adaptor σ subunits[39]. Since AP-1 depletion does not block Vpu function, we wondered whether Vpu interaction with the clathrin machinery might also occur through AP-2. We therefore analyzed the precipitations from cells expressing tetherin Y6,8A for the AP-2α adaptin subunit (Figs 6A, 6B and S7B). Surprisingly this could also be detected with the wild-type protein, but was absent for all the mutants, indicating ExxxLV also regulates this interaction. Thus, Vpu interacts promiscuously with both major cellular clathrin adaptors in a manner dependent on its ability to bind to tetherin. This is likely to account for why individual adaptor knockdowns fail to block Vpu function, and suggest that AP-2 might represent a compensatory clathrin-dependent trafficking mechanism for counteracting tetherin.

**Fig 7:**
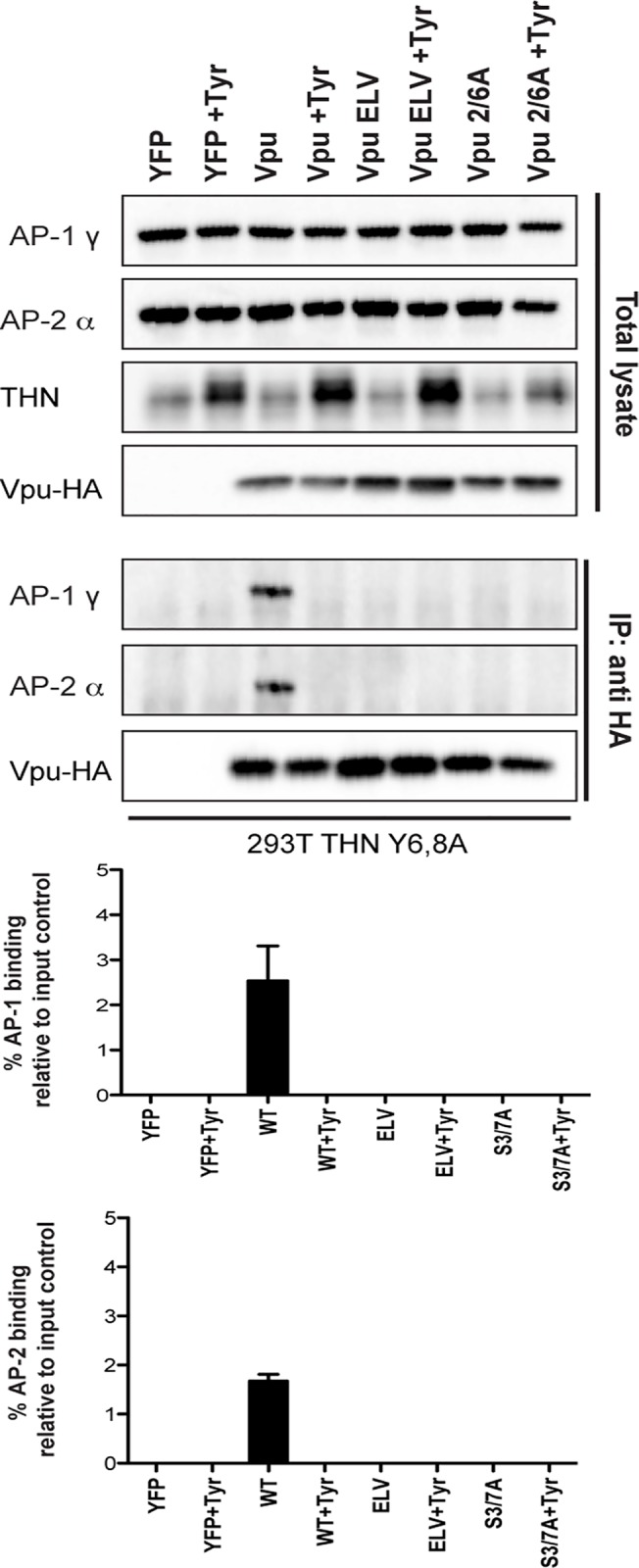
Vpu interaction with clathrin adaptors AP-1 and AP-2 is abrogated following treatment with CKII inhibitor, Tyrphostin. 293T tetherin Y6,8A cells were transfected with pCR3.1 Vpu-HA, Vpu A14L/W22A-HA, Vpu ELV-HA, Vpu 2/6A-HA, Vpu LILI-HA or Vpu NE-HA mutants. 24h post-transfection cells were treated with DMSO or 50 μM Tyrphostin. Cells were lysed and cross-linked, 48 h post transfection, using PFA (0.05% w/v) and immunoprecipitated with anti-HA antibody. Total cell lysates and precipitates were subjected to SDS-PAGE and analyzed by Western blotting for Vpu-HA, tetherin, AP-1γ or AP-2α. Panels are of representative experiments. Histograms represent quantification of the relative AP-1 or AP-2 binding normalized to input control. Error bars represent the standard deviation of three independent experiments.

Finally, to provide direct evidence that it was phosphorylation of Vpu that permitted AP1/AP2 interactions, we repeated these immunoprecipitations in 293T tetherin Y6,8A cells treated with Tyrphostin (Fig 7). Under these conditions the ability of wildtype Vpu to interact with AP1 or AP2 was abolished, indicating that CKII-mediated phosphorylation for Vpu is required for recruitment of clathrin transport machinery.

## Discussion

In this study we have re-evaluated discrepancies in the literature regarding the role of SCF^βTRCP1/2^ and ESCRT in Vpu-mediated tetherin degradation and antagonism of its physical antiviral activity. We find that whilst essential for the former, they are dispensable for the latter in HIV-1 infected cells. We further show that phospho-serine mutants of Vpu have a distinct phenotype, displaying defects in tetherin antagonism because they cannot engage with clathrin-dependent trafficking pathways. We demonstrate that *in cellulo* Vpu/tetherin TM interactions induce Vpu binding to either clathrin adaptors AP-1 or AP-2. This interaction requires the ExxxLV trafficking motif, validating the recent structural study [39]. Importantly, phosphomutants of Vpu are also defective for clathrin adaptor engagement, implying that CKII-mediated phosphorylation not only regulates SCF^βTRCP1/2^ recruitment, but also regulates Vpu trafficking. Together these data clarify the role of the Vpu DSGNES motif in tetherin counteraction and provide strong evidence that sorting of Vpu/tetherin complexes into clathrin-rich domains of the endocytic pathway is the critical event in efficient tetherin antagonism. Furthermore, the observation that Vpu can interact both with AP-1 or AP-2 suggests a redundancy in adaptor protein requirement for tetherin counteraction that provides a plausible explanation for why depletion of either AP-1 or AP-2 is not sufficient to compromise Vpu function[36]. Thus potentially, tetherin/Vpu complexes that escape AP-1 in the TGN, and which traffic to the PM, can be retrieved by AP-2. Such a model would also rationalize why in some cases tetherin counteraction by Vpu can be observed with minimal evidence of surface downregulation [18,48].

Much of the discrepant literature regarding the mechanism of Vpu-mediated tetherin antagonism comes from experiments where tetherin, provirus and/or Vpu are transiently transfected into cells. Whilst these experiments are useful for understanding much of the biology of tetherin/HIV interactions, they are prone to artifacts when interpreting the cell biology and importance of Vpu-mediated degradation. Overexpression of tetherin or Vpu at non-physiological levels has been shown to induce ER-associated degradation [30]. This is not observed in infected cells, where tetherin is degraded in endosomes. Also, because of the nature of transient transfections, there is a huge variability of expression levels of the transfected components between cells within the culture. Under these conditions strong blocks to degradation may lead to tetherin accumulation, and an overwhelming of the endosomal system, giving the appearance of a direct inhibition of counteraction. By infecting tetherin-expressing cells at relevant multiplicities of infection, to ensure each cell has on average one productive infection event, these issues can be mitigated and this has allowed us to separate the requirement of the phospho-serine motif in counteraction from the recruitment of SCF^βTRCP1/2^ and the ESCRT machinery for degradation.

Our *in cellulo* data validates the structural and biochemical studies by Jia et al [39], in which AP-1 interaction requires the ExxxLV motif that occupies the acidic-dileucine binding site in AP-1σ. We also provide evidence that in cells, this motif can also bind to AP-2. Furthermore, the phenotype of our LI/LI mutant is consistent with the proposed non-canonical interaction of R44/L45 with AP-1μ suggested by densities in the crystal. However, because the constructs used by the authors to determine the structural requirements for AP-1/tetherin/Vpu interaction required artificial Vpu/tetherin fusions, they may not faithfully represent how AP-1 is initially recruited. Thus, the requirements for the DSGNES and Vpu/tetherin transmembrane domain interactions that we have uncovered in cells were not previously observed.

**Fig 8:**
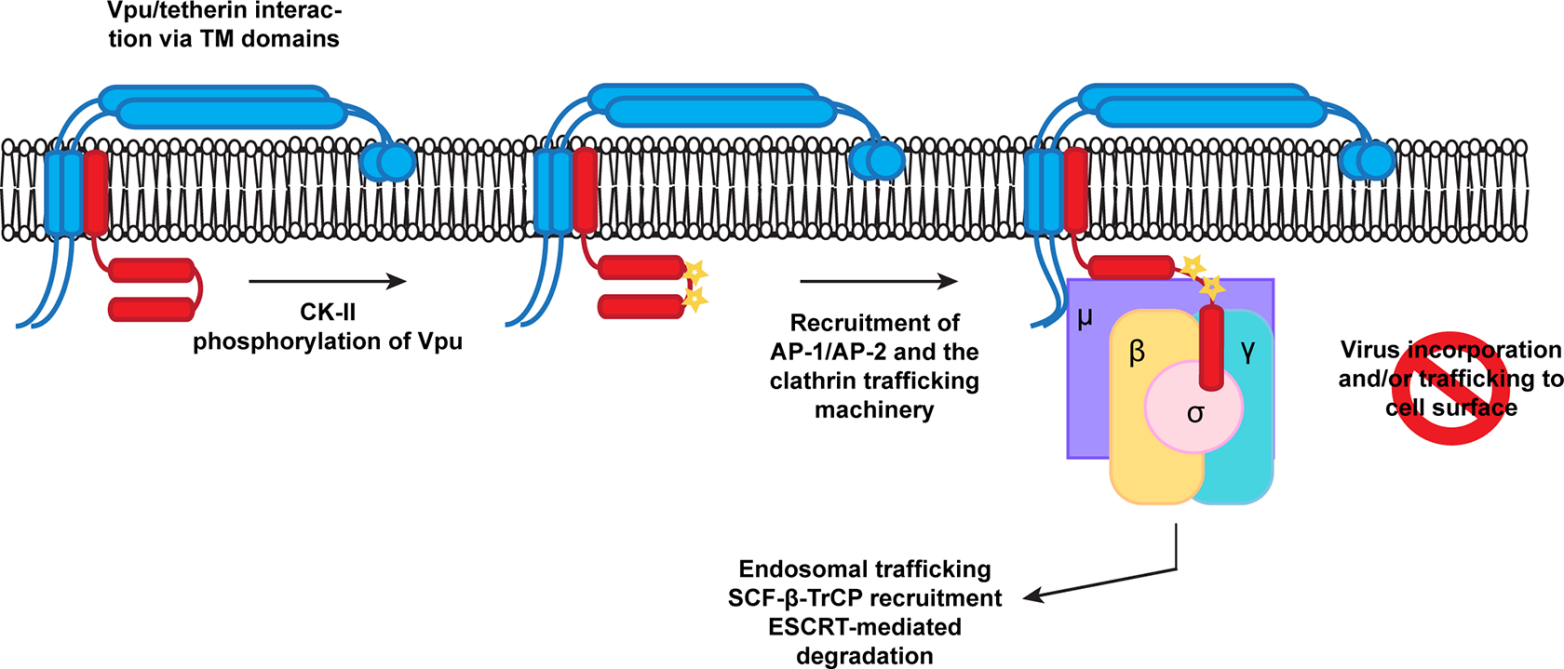
A proposed model for Vpu engagement of clathrin adaptors during tetherin counteraction. Vpu and tetherin interactions via TM/TM domain interactions and casein kinase II phosphorylation promote Vpu recruitment of AP-1 or AP-2. This allows the EXXXLV motif to bind to the σ subunit, and potentially through non-canonical interactions between its first alpha helix with the AP-1 or 2 μ subunits. In addition the YCRV motif in tetherin binds to the AP1μ. Thus tetherin/Vpu complexes are sorted into clathrin rich domains of the TGN or PM for subsequent trafficking and ubiquitination.

We propose a model whereby phosphorylation of Vpu regulates the AP interaction with the ELV motif (Fig 8). Whilst we cannot formally rule out that the phosphoserine directly contributes to AP-1 interaction itself, the lack of a significant additive phenotype in terms of virus release and AP-1 interaction makes this the most consistent explanation of our data. Furthermore there is precedence for phosphorylation upstream of certain acidic dileucine motifs interactions with the clathrin transport machinery [43]. In particular, a CKII phosphorylation upstream of a non-canonical RDDHLL site in the cation-independent mannose-6-phosphate receptor regulates its interaction with AP1. Another context-dependent feature of acidic dileucine signals is an adjacent acidic patch [37]. Interestingly, this feature is present in HIV-1 Vpu. Furthermore, the laboratory strain NL4.3 Vpu, which has a reduced anti-tetherin activity compared to most primary isolates, has a shorter acidic patch between the DSGNES and ExxxLV motifs [34]. The requirement for TM interactions in addition to the phospho-serines in “priming” Vpu for clathrin adaptor interaction would imply that tetherin binding contributes to conformational changes that are required for antagonism. Since β-TrCP binding does not require the presence of tetherin (or CD4), phosphorylation must be an independent event. However, whether β-TrCP and AP-1/2 binding can occur simultaneously or are mutually exclusive is unknown. Another interesting point to note is that the LI/LI mutation is more severely compromised than either the 2/6 or the ELV mutations in some contexts. As it also compromises AP binding, the non-canonical interaction of the R45,L46 with AP-1μ may also play an essential contextual role in positioning the ELV motif. This interaction may also explain why the residual activities of 2/6 and ELV mutations are sensitive to clathrin depletion.

Structural information on the Vpu cytoplasmic tail is limited at present. Partial NMR structures in solution and associated with lipids have been determined [49–52]. In a lipid environment, the ExxxLV is embedded within helix 2 of the cytoplasmic tail [52], but adopts an extended conformation in solution [51]. To bind to AP-1, the ExxxLV site cannot be helical. However, the lipid-associated structure has a very interesting feature: a highly conserved C-terminal tryptophan residue appears to pack against the DSGNES, almost as if locking the structure. Mutations in the W residue have context-dependent defects in tetherin antagonism depending on the Vpu used [34,53]. Importantly, NMR studies on the effects of serine phosphorylation suggests that it leads to conformational changes within the C-terminal region of the Vpu cytoplasmic tail that promotes βTRCP binding. In some studies [49,50], but not others [54], these conformational changes are consistent with an opening up of the ELV site. However, all these studies have thusfar been performed in the absence of target binding using soluble Vpu cytoplasmic tails, and so how representative they are of the wildtype protein is unclear. Furthermore, upregulation of SCYL2, a clathrin associated protein that modulates protein phosphatase 2A (PP2A), induces Vpu de-phosphorylation and reduces tetherin antagonism [55]. Thus, there is scope for regulated phosphorylation and subsequent dephosphorylation cycles in regulation of Vpu activity. We suggest that this would occur at the level of clathrin-dependent transport rather than SCF^βTRCP1/2^ interactions.

There is much indirect evidence consistent with AP-1 being the major clathrin adaptor used by Vpu. The block to tetherin transport to the surface, the predominant localization to the TGN, and of course the recent structure discussed above [33,35,36,39]. However, AP-1 knockdown is difficult to efficiently achieve and does not compromise Vpu function [36]. AP-1 has multiple orthologs for some of its subunits, and there is potential redundancy in the adaptor machinery allowing the cell to compensate for its absence [37]. Our observation that Vpu can interact also with AP-2 in an ExxxLV-dependent manner is therefore an important observation for several reasons. Firstly, it suggests that Vpu is promiscuous and if one adaptor is compromised, another can be used, explaining why neither AP-1 nor AP-2 have been unambiguously identified as Vpu cofactors [25,36]. Secondly, it might explain why in some studies, Vpu has been observed to induce a weak enhancement of tetherin endocytosis [35]. Artificial tetherin/Vpu linked chimeric proteins are excluded from budding virions, and this is dependent on the ExxxLV motif [18], which would be consistent with anchoring by AP-2 into clathrin-rich domains at the plasma membrane. The YDYCRV motif of tetherin cannot interact with AP-2μ as a YXXθ signal because of a steric clash of Y6 in the binding pocket [39]. The YDYCRV motif is essential for the “slow”, AP-1-dependent recycling of tetherin to the PM via the TGN [17,33]. Therefore, Vpu is likely to meet the majority of its target (newly synthesized and recycling tetherin) in the TGN. Since AP-1 has been proposed to regulate bidirectional traffic between the TGN and endosomal compartments [37], AP-1 is likely to be the major player in tetherin counteraction. However, the ExxxLV motif is dominant over the tetherin recycling motif [36]. Therefore we would predict that tetherin/Vpu complexes that escape re-routing in the TGN and make it to the PM would be excluded from virions and AP-2 would promote their endocytosis, much in the same way that SIV Nef proteins and HIV-2 envelopes antagonize tetherin [6,38,56]. More importantly, it also accounts for why Vpu still has some activity against the short tetherin isoform without appreciable cell surface downregulation [13,40]. The relative role of AP-1 and AP-2 will reflect the kinetics of their respective activities in different cell types. We suggest the combination of some or all of the above accounts for the variable importance that downregulation of tetherin from the PM has been given to its antagonism. The requirement for the ExxxLV and DSGNES motifs is not absolute when tetherin levels are low. At higher expression levels, such as upon IFN treatment of primary CD4+ T cells, they become essential for tetherin antagonism [36]. This residual function requires tetherin’s sorting motif, suggestive of competition between the clathrin-dependent trafficking and virion retention. Tetherin/Vpu interaction may simply tip this balance, reducing tetherin partitioning into virions sufficiently when its expression levels are low. It is this that we propose to augment via our clathrin box fusion rescue, locking the tetherin/Vpu complex into clathrin-rich domains in the recycling pathway from where they cannot be transited to the PM.

Decoupling tetherin degradation (which amongst primate lentiviruses is so far peculiar to HIV-1 group M Vpu) from subversion of trafficking (counteraction) suggests that the importance of the former might reflect downstream consequences of tetherin restriction. Enhanced antagonism of the long tetherin isoform by Vpu could be required because of its signal transduction or its ability to deliver retained virions to endosomes [14,40]. Our data shows that in stable tetherin expressing cells, STS mutations impart little resistance to Vpu and that they are still sensitive to Vpu-clathrin box fusions. Since neither LI/LI nor ELV mutations block binding of Vpu to β-TrCP or tetherin, ubiquitination may still occur on serine and threonine residues. However, its effect is likely to be subsequent to antagonism by clathrin-dependent mis-trafficking. Strong reduction of tetherin at the cell surface by Vpu coupled to endosomal degradation would therefore be a potent way of suppressing signal transduction, or blocking the routing of virions for degradation where they may encounter other host pattern recognition receptors or antigen processing machinery. These will be important attributes to maintain *in vivo* without necessarily being essential for physical antagonism of tetherin.

## Materials and Methods

### Cells, plasmids and reagents

HEK293T cells were obtained from ATCC (American Tissue Culture Collection). 293T tetherin cell lines stably expressing human tetherin and mutants were previously described [4,57]. The HeLa-TZMbl reporter cell line, was kindly provided by John Kappes through the NIH AIDS Reagents Repository Program (ARRP). Cells were maintained in Dulbecco’s modified Eagle medium (DMEM) supplemented with 10% fetal calf serum and Gentamycin (Invitrogen, UK). Wildtype HIV-1 NL4.3 (obtained from NIH-ARRP), a Vpu-defective counterpart and a codon optimized pCR3.1 Vpu-HA has been described previously [58]. The Vpu A14L/W22A, ELV, 2/6A, LILI and NE mutants in pCR3.1 Vpu-HA and in the NL4.3 proviral genome were generated by Quick-change site-directed mutagenesis PCR according to standard protocols using Phusion-II polymerase (New England Biolabs). A codon-optimised version of the previously described primary wild-type HIV-1 Vpu 2_87 [34] was HA-tagged and cloned into pCR3.1. The Vpu A15L/W23A, ELV, 2/6A, LILI and NE mutants were generated in pCR3.1 Vpu 2_87-HA by Quick-change site-directed mutagenesis as described above. Consensus B codon-optimised Vpu-myc-BirA-R188G fusion was synthesized (Life Technologies) and cloned into the lentiviral vector pAIP (kindly provided by A Cimarelli). The Vpu A15L/W23A, ELV, 2/6A and LILI mutants were generated by Quick-change site-directed mutagenesis as described above. The pCR3.1 myc-β-TrCP2 was previously described by [36] and the pCR3.1 myc-HRS expression vector was kindly provided by Juan Martin-Serrano [59].

Primary human CD4+ T cells were isolated from fresh venous blood drawn from healthy volunteers. CD4+ T cells were purified from total peripheral blood mononuclear cells (PBMC) isolated by lymphoprep (AXIS-SHIELD) gradient centrifugation using a CD4+ T cell Dynabeads isolation kit (Invitrogen). T cells were then activated for 48 h using anti-CD3/anti-CD28 magnetic beads (Invitrogen). The beads were then removed cells were then maintained in rhIL-2 (20 U/ml) (Roche).

### Production of VSV-G pseudotyped viral stocks

For full-length HIV-1 WT, HIV-1 ΔVpu, HIV-1 Vpu LILI, HIV-1 Vpu ELV, HIV-1 Vpu LILI/ELV, HIV-1 Vpu 2/6A, HIV-1 Vpu 2/6A/ELV virus stocks pseudotyped with the Vesicular Stomatitis Virus Glycoprotein (VSV-G), 293T cells were transfected with 2 μg of proviral plasmid in combination with 200 ng of pCMV VSV-G. 48 hours post-transfection, the supernatant containing virions was harvested and endpoint titers were determined on HeLa-TZMbl cells as described previously [3].

### Virus release assay

For virus release assays using transient transfection, subconfluent 293T cells or derivatives were plated in 24 well plates and transfected with 500 ng of NL4.3 proviral plasmid, in combination with increasing concentrations of tetherin (0 ng, 25 ng, 50 ng and 100 ng) and fixed 25 ng of Vpu-HA or mutants using 1 μg/ml polyethyleneimine (Polysciences). Medium was replaced 8 hours post-transfection and cells and supernatants were harvested after 48 hours. The infectivity of viral supernatants was determined by infecting HeLa-TZMbl and assayed for β-galactosidase activity as previously described [36]. For biochemical analysis of physical virus particle release, supernatants were filtered (0.22 μm) (Merck Millipore) and pelleted through a 20% sucrose/ PBS cushion at 20,000 x g for 90 min at 4°C. Virion and cell lysates were subjected to SDS-PAGE and Western blotted for rabbit anti-HSP90 (Santa Cruz Biotechnologies), HIV-1 p24CA (monoclonal antibody 183-H12-5C; kindly provided by B Chesebro through the NIH ARRP), monoclonal mouse anti-HA.11 (Covance), polyclonal rabbit anti-HA (Rockland) and/ or Vpu (rabbit polyclonal; kindly provided by K. Strebel through the NIH ARRP [60]. For CK-II inhibition, we used Tyrphostin AG1112 (Sigma) reconstituted in DMSO at a concentration of 50 μM. Where indicated, Phos-tag (Wako Chemicals, Japan) and MnCl_2_ (Sigma) were added to the composition of 8% polyacrylamide gels to induce mobility shifts in phosphorylated proteins, to final concentrations of 25 μM and 50 μM, respectively.

### Tetherin degradation assay

1.5 x 10^5^ 293T tetherin cells were infected with VSV-G-pseudotyped HIV-1 WT, HIV-1 ΔVpu, HIV-1 Vpu LILI, HIV-1 Vpu ELV or HIV-1 Vpu 2/6A at an MOI of 2. The medium was replaced 4 hours after infection. 48 hours post infection cell lysates were harvested and subjected to SDS-PAGE and Western blotted for rabbit anti-HSP90 (Santa Cruz Biotechnologies) and polyclonal rabbit anti-tetherin antibody (kindly provided by K Strebel through the NIH ARRP) [48], and processed as described above.

### siRNA mediated protein knockdown

293T tetherin cells were seeded at a density of 2 x 10^5^ cells per well in a 12 well plate. After 6 hours, the first transfection was performed. For each well, 2 μl Dharmafect (Thermo Scientific) was added to 98 μl of Opti-MEM (Life Technologies), this solution was added to 5 μl of 20 μM siRNA in 95 μl of Opti-MEM according to manufactures protocol. For HRS knockdown, siRNA oligonucleotide against HGS targeting the CCGGAACGAGCCCAAGTACAA sequence (Qiagen) was used. For UBAP1 knockdown, siRNA oligonucleotide against UBAP1 targeting CTCGACTATCTCTTTGCACAT (Qiagen) was used. For TSG101 knockdown, siRNA oligonucleotide sequence CCUCCAGUCUUCUCUCGUCUU (Thermo Scientific) was used. For β-TrCP1 and 2 knockdown, SMARTpool siRNA against human BTRC and FBXW11 (Thermo Scientific) were used. A non-targeting siRNA was used as control (Thermo Scientific). The cells were re-seeded into a 24 well plate on day 2 and a second transfection was performed according to manufactures protocol. The cells were infected 3 hours post transfection with VSV-G-pseudotyped HIV-1 WT, HIV-1 ΔVpu at an MOI of 0.8. The infectivity of viral supernatants was determined by infecting HeLa-TZMbl as described above. Cell lysates and viral particles were subjected to SDS-PAGE, and Western blot assays were performed using a rabbit polyclonal anti-HRS (HGS) antibody (Millipore), a polyclonal rabbit anti-UBAP1 antibody (Proteintech) and a monoclonal mouse anti-TSG101 antibody (Abcam).

### Flow cytometry

HeLa-TZMbl cells were transfected with 400 ng of pCR3.1 GFP and 400 ng of pCR3.1 Vpu-HA or indicated mutants. 48 hours post transfection the cells were harvested and stained for surface tetherin using a monoclonal anti-BST2 IgG2a antibody (Abnova) and a goat-anti-mouse IgG2a-Alexa633 conjugated secondary antibody (Molecular Probes, Invitrogen, UK). Tetherin expression on GFP positive cells was then analyzed using a BD FACSCanto II flow-cytometer (Becton Dickinson) and the FlowJo software.

### Immunoprecipitation

For Vpu/HRS coIP, 293T tetherin cells were transfected with 700 ng of pCR3.1 myc-HRS or indicated mutants/truncations in combination with pCR3.1 Vpu-HA or indicated mutant or pCR3.1 GFP expression plasmids. 48 hours post transfection the cells were lysed in buffer containing 50 mM Tris pH 7.4, 150 mM NaCl, 200 μM sodium ortho-vanadate, 5 mM NEM, complete protease inhibitors (Roche) and 1% digitonin. After removal of the nuclei, the supernatants were immunoprecipitated with 5 μg/ml monoclonal mouse anti-myc antibody previously described (Kueck and Neil, 2012). Western blot assays were performed using a polyclonal rabbit anti-HA antibody (Rockland) and rabbit polyclonal anti-HRS (HGS) antibody (Millipore). For Vpu/tetherin coIP, 293T cells were transfected twice over 48 hours with siRNA oligonucleotide against UBAP1 targeting CTCGACTATCTCTTTGCACAT or Non-targeting siRNA was used as control (Dharmacon). The cells were then infected with VSV-G-pseudotyped HIV-1 WT, HIV-1 ΔVpu, HIV-1 Vpu LILI or HIV-1 Vpu A14L W22A at an MOI of 2. 48 hours post infection the cells were lysed on ice for 30 min in buffer containing 50 mM Tris pH 7.4, 150 mM NaCl, complete protease inhibitors (Roche) and 1% digitonin (Calbiochem). Immunoprecipitation was performed as previously described [36] and Western blot assays were performed using a rabbit anti-Vpu antibody polyclonal rabbit anti-tetherin antibody and polyclonal rabbit anti-UBAP1 antibody (Proteintech), and visualized by ImageQuant using corresponding HRP-linked secondary antibodies (New England Biolabs, UK).

### Immunofluorescence

Hela cells were grown on coverslips, transfected with 50 ng of pCR3.1 Vpu-HA or indicated mutant. 16 hours later cells were fixed in 4% paraformaldehyde/ PBS, washed with 10 mM glycine/ PBS, and permeabilized in 1% bovine serum albumin/ 0.1% Triton-X100/ PBS for 15 min. Cells were stained using anti-rabbit polyclonal HA antibody (Rockland) in combination with sheep anti-human TGN46 (AbD Serotec), followed by the appropriate secondary antibodies conjugated to Alexa 488 or 594 fluorophores (Molecular Probes, Invitrogen). Cells were mounted on glass slides using ProLong AntiFade- 4’,6-diamidino-2-phenylindole (DAPI) mounting solution (Molecular Probes, Invitrogen) and images were captured with a Nikon ESCLIPSE Ti inverted microscope. Z stacks were taken of all cells, images deconvolved using AutoQuant X3 and analyzed using the ImageJ software.

### Cross-linking IP

293T, 293T tetherin, 293T tetherin Y6,8A or 293 Rhesus tetherin cells were transfected with 8 μg GFP expression construct, pCR3.1 Vpu-HA or mutant thereof. Transfection media was changed 6 hours post transfection and cells incubated with 50 nM concanamycin. In the case of CKII inhibitor treatment, cells were treated with 50 μM final Tyrphostin 24 hours prior to harvesting. 48 hours post transfection, cells were trypsinised and washed in PBS. Cells were cross-linked with 0.05% HCHO/PBS for 10 min at 37°C. The cross-linking reaction was then quenched by incubating cells in 0.25 M glycine for 5 min. Cells were washed once in PBS before resuspension in lysis buffer (10 mM Hepes pH 7, 150 mM NaCl, 6 mM MgCl2, 2 mM DTT, 10% glycerol, 0.5% NP40, 200 μM sodium orthvanadate and 1x Complete protease inhibitors (Roche)). Cells were lysed on ice for 10 min followed by repeated sonication (3 x 10 s cycles with 20 s rests). The cell lysates were clarified by centrifugation at 1000 x g for 10 min and supernatants were immunoprecipitated with 5 μg/ml mouse monoclonal anti-HA.11 antibody (Covance) or rabbit polyclonal anti-HA antibody (Rockland) on Dynabeads protein G beads (Life Technologies) for 4 hours at 4°C. Beads were collected post incubation and washed 5 times in lysis buffer before cross-links were reversed in 1% SDS, 10 mM EDTA and 5 mm DTT at 65°C for 45 min. Western blot assays were performed using rabbit polyclonal anti-HA antibody (Rockland), polyclonal rabbit anti-tetherin antibody, mouse monoclonal anti-HA.11 antibody, mouse monoclonal anti-AP-1γ1 antibody (Sigma) and mouse monoclonal anti-AP-2α antibody (Sigma). Vpu/β-TrCP2 cross-linking IP was previously described by [36].

### Affinity purification of biotinylated proteins

[61] 293T or 293T tetherin cells were transiently transfected with 8 μg empty BirA vector, Vpu-myc-BirA or relevant mutant constructs using polyethylenimine (PEI). Cells were incubated for 8 hours prior to changing medium and treated overnight with 100 nM Concanamycin A (Invitrogen) and 150 μM biotin (Invitrogen). Cells were washed twice in PBS and lysed in 1 ml lysis buffer (50 mM Tris pH 7.4, 500 mM NaCl, 0.4% SDS, 5 mM EDTA, 1 mM DTT and 1x Complete protease inhibitor (Roche)) before sonication. Triton-X-100 was added to a final concentration of 2% before further sonication and an equal volume of 50 mM Tris pH 7.4 was added to the cell lysates before clarification at 14,000 rpm for 5 minutes. Supernatants were incubated with 200 μl avidin agarose (Pierce) for 4 hours at 4°C. Beads were collected and washed four times in 1 ml lysis buffer before resuspension in 100 μl Laemmli-SDS sample buffer supplemented with free biotin. Cell lysates and precipitates were analysed by Western blot using HRP-conjugated streptavidin (Invitrogen), mouse monoclonal anti-myc antibody (Covance), rabbit monoclonal β-TrCP antibody (Cell signaling Technology) and mouse monoclonal anti-AP-1γ1 antibody (Sigma).

### Ethical information

Permission to isolate primary human CD4+ T cells from healthy consenting donors was provided by the KCL Infectious Disease BioBank Local Research Ethics Committee, reference SN-1/6/7/9.

## Supporting Information

S1 Fig Vpu/HRS interaction is dependent on residues in the DUIM of HRS that bind ubiquitin. (A) Schematic representation of HRS C-terminal truncations. (B) 293T cells were transfected with pCR3.1 Vpu-HA in combination with a pCR3.1 myc-HRS or myc-HRS truncation expression vector. 48 hours post transfection cells were lysed and immunoprecipitated with anti-myc antibody. Total cell lysates and precipitates were subjected to SDS-PAGE and analysed by Western blotting for myc-HRS and Vpu, and analyzed by ImageQuant. Asterisk: anti-HA antibody heavy chain (C) Immunoprecipitation was performed as in (B) but with myc-HRS W25A L29D and myc-HRS A266Q A268Q mutants.
(TIF)

S2 Fig Vpu LI/LI mutant exhibits impaired tetherin counteractivity. (A) LogoPlots of the first alpha helix of the Vpu cytoplasmic tail from HIV-1 subgroup M clades A, B, C, D, G and H generated from sequences obtained from the Los Alamos database (www.hiv.lanl.gov). (B) 293T cells were transfected with NL4.3 HIV-1 WT, ΔVpu, Vpu LILI, Vpu ELV or Vpu 2/6A mutant together with increasing concentrations of pCR3.1 tetherin-HA expression plasmid. Cell lysates and sucrose purified viral supernatants from 50 ng tetherin input were subjected to SDS-PAGE and analyzed by Western blotting for HSP90, HIV-1 p24CA and Vpu, and analyzed by LiCor quantitative imager. Asterisk: non-specific band. (C) Infectivity of viral supernatants was assayed on HeLa-TZMbl reporter cells. Infectious virus release was plotted as β-galactosidase activity in relative light units (RLU). Error bars represent the standard deviation of three independent experiments. (D) HeLa-TZMbl cells were co-transfected with pCR3.1 Vpu-HA or indicated mutant and a GFP expression vector. Cell-surface tetherin levels were analysed 48 hours post transfection by flow cytometry. GFP positive cells were gated and tetherin levels (solid lines) were compared to un-transfected cells or transfected with indicated Vpu (dotted lines). Numbers indicate median fluorescence intensities of endogenous tetherin surface levels. The solid peak in the upper histogram in the middle of the panel represents binding of the isotype control. (E) 293T tetherin expressing cells were transfected twice over a 48 hour period with siRNA oligonucleotides directed against UBAP1 or non-targeting control. The cells were then infected with HIV-1 WT, HIV-1 Vpu LILI, HIV-1 Vpu A14L W22A or HIV-1 ΔVpu at an MOI of 2. 48 hours later, cells were lysed and immunoprecipitated with anti-tetherin antibody. Total cell lysates and precipitates were subjected to SDS-PAGE and analyzed by Western blotting for tetherin, UBAP1 and Vpu, and analyzed by ImageQuant.
(TIF)

S3 Fig Further Vpu mutants show similar phenotypes in infected primary CD4+ T cells and in 293T cells. (A-B) 293T, 293T tetherin or Y6,8A tetherin cells were infected with VSV-G pseudotyped NL4.3 wt or mutant virus at an MOI of 0.8. (A) 48 hours post infection viral supernatants were assayed for infectivity using HeLa-TZMbl reporter cells as in Fig 1. Error bars represent the standard deviation of three independent experiments. (B) Cell lysates and sucrose purified viral supernatants were subjected to SDS-PAGE and analyzed by Western blotting as in Fig 1. (C) Primary human CD4+ T cells were infected with the indicated HIV-1 mutant at an MOI of 0.8. 16 h later the cells were treated or not with 5000 U/ml universal type-I interferon. Cell lysates and viral supernatants were harvested a further 24 h later and analyzed for infectivity on HeLa-TZMbl cells (A) or physical particle yield and cellular viral expression by quantitative Western blotting.
(TIF)

S4 Fig A primary isolate Vpu allele and its mutants exhibit a comparable phenotype to NL4.3 Vpu. (A) 293T tetherin cells were transfected with NL4.3 ΔVpu proviral plasmid in combination with YFP expression vector and pCR3.1 2_87 Vpu or mutants thereof. 48 hours post transfection infectivity of viral supernatants was determined on HeLa-TZMbl cells as in Fig 1. (B) Cell lysates and pelleted supernatant virions from (A) were harvested and subjected to SDS-PAGE and analyzed by Western blotting for HIV-1 p24CA, Vpu and HSP90, and analyzed by LiCor quantitative imager. (C) Hela cells were transfected with 100 ng of pCR3.1 2_87 Vpu-HA or indicated mutants. 16 hours post transfection cells were fixed and stained for HA (green) and the TGN marker TGN46 (red) and examined by widefield fluorescent microscopy. Panels are of representative examples. Bars = 10 μm. (D) Z stacks were taken of all cells (n = 15), images were deconvolved using the AutoQuant X3 software and Pearson’s correlations were calculated for all Z stacks using ImageJ. Results were analyzed by unpaired 2-tailed t-test—*** P = 10–5 or lower.
(TIF)

S5 Fig Clathrin binding restores the tetherin downregulation capacity of Vpu mutants. (A to D) HeLa-TZMbl cells were co-transfected with pCR3.1 Vpu-HA or indicated mutant and a GFP expression vector. Cell-surface tetherin levels were analyzed 48 hours post transfection by flow cytometry. GFP positive cells were gated and tetherin levels (solid lines) were compared to un-transfected cells or transfected with indicated Vpu (dotted lines). Numbers indicate median fluorescence intensities of endogenous tetherin surface levels. The solid peak in the upper histogram in the middle of the panel represents binding of the isotype control.
(TIF)

S6 Fig Proximity-based biotin ligase assay suggests Vpu/AP-1 interaction. (A) Schematic representation of proximity-based biotin ligase assay. (B) 293T tetherin cells were transfected with NL4.3 ΔVpu proviral plasmid in combination with YFP expression vector and pCR3.1 Vpu or indicated mutant thereof. 48 hours post transfection infectivity of viral supernatants was determined on HeLa-TZMbl cells as in Fig 1. (C) 293T or 293T tetherin cells were transfected with Vpu-myc-BirA, B Vpu ELV-myc-BirA, B Vpu A15L/W23A-myc-BirA, B Vpu S3/7A- myc-BirA (phospho-mutant), B Vpu LILI-myc-BirA or empty vector control. 6 hours post transfection, cells were treated with 100 nM concanamycin A in the presence of 150 μM free biotin. 16 hours later, cells were washed, lysed, sonicated and biotinylated proteins were recovered on streptavidin-conjugated beads and analysed by Western blot for avidin, Vpu-myc-BirA, β-TrCP or AP-1 γ. Asterisk: Vpu-myc-BirA band.
(TIF)

S7 Fig Vpu binding to clathrin adaptors AP-1 and AP-2 is dependent on tetherin binding (A) and binding to AP-1 and AP-2 is conserved by primary Vpu 2_87 in tetherin expressing cells (B). (A) 293T cells were transfected with pCR3.1 Vpu-HA, Vpu ELV-HA, Vpu 3/7A-HA, Vpu LILI-HA or Vpu NE-HA mutants. (B) 293T tetherin Y6,8A were transfected with pCR3.1 Vpu-HA, Vpu A15L/W23A-HA, Vpu ELV-HA, Vpu 3/7A-HA, Vpu LILI-HA or Vpu NE-HA mutants. 48 h post transfection, cells were lysed and cross-linked using PFA (0.05% w/v) and immunoprecipitated with anti-HA antibody. Total cell lysates and precipitates were subjected to SDS-PAGE and analyzed by Western blotting for Vpu-HA, AP-1 Ɣ or AP-2 α. Panels are of representative experiments. Histograms represent quantification of the relative AP-1 or AP-2 binding normalized to input control. Error bars represent the standard deviation of three independent experiments.
(TIF)
