# Supplementary figures and images for: Serine Phosphorylation of HIV-1 Vpu and Its Binding to Tetherin Regulates Interaction with Clathrin Adaptors
Source: PLoS Pathog. 2015 Aug 28;11(8):e1005141. doi: 10.1371/journal.ppat.1005141 (PMC4552633; doi:10.1371/journal.ppat.1005141)

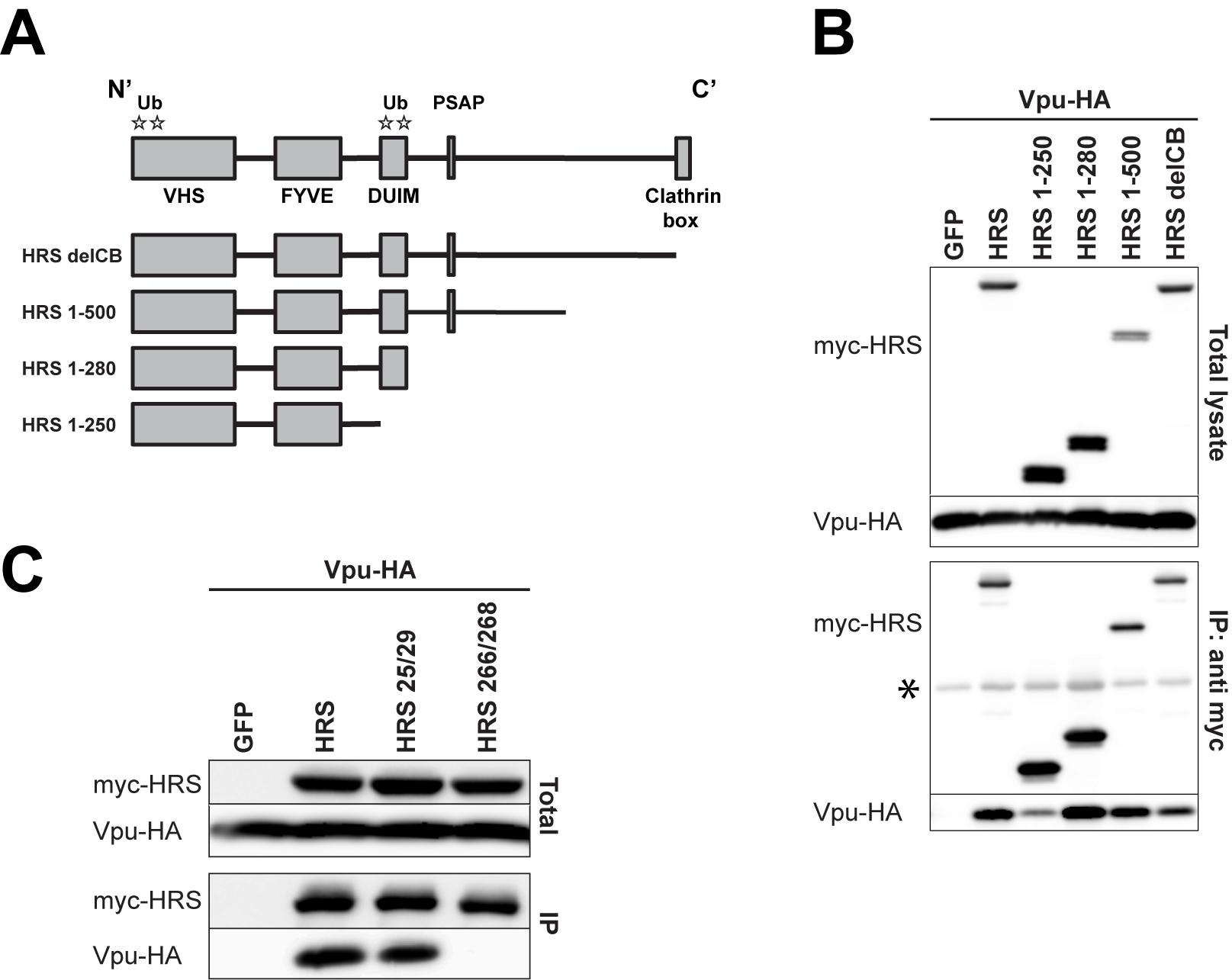

Supplement: S1 Fig — (A) Schematic representation of HRS C-terminal truncations. (B) 293T cells were transfected with pCR3.1 Vpu-HA in combination with a pCR3.1 myc-HRS or myc-HRS truncation expression vector. 48 hours post transfection cells were lysed and immunoprecipitated with anti-myc antibody. Total cell lysates and precipitates were subjected to SDS-PAGE and analysed by Western blotting for myc-HRS and Vpu, and analyzed by ImageQuant. Asterisk: anti-HA antibody heavy chain (C) Immunoprecipitation was performed as in (B) but with myc-HRS W25A L29D and myc-HRS A266Q A268Q mutants. (TIF) [file ppat.1005141.s001.tif]

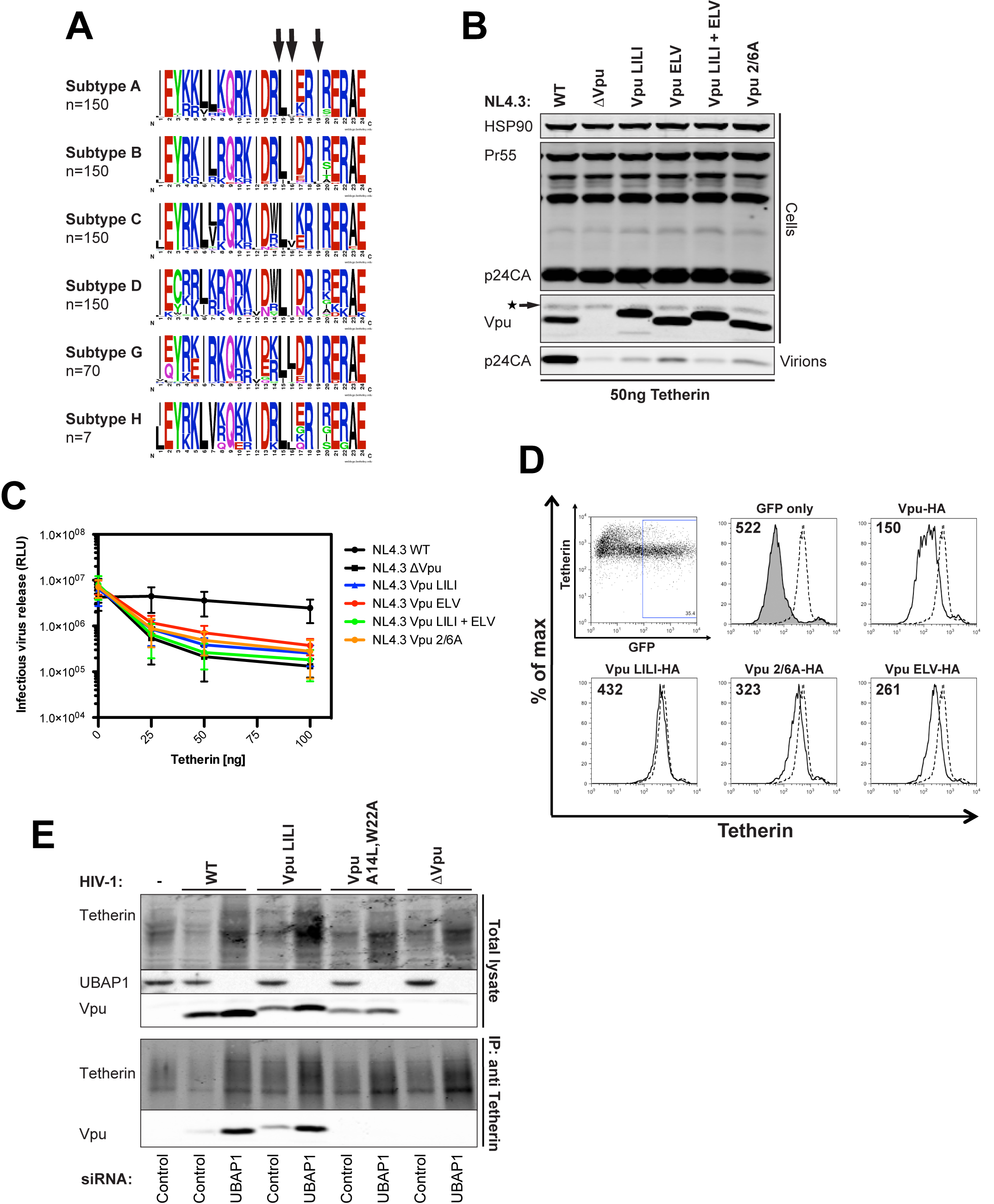

Supplement: S2 Fig — (A) LogoPlots of the first alpha helix of the Vpu cytoplasmic tail from HIV-1 subgroup M clades A, B, C, D, G and H generated from sequences obtained from the Los Alamos database (www.hiv.lanl.gov). (B) 293T cells were transfected with NL4.3 HIV-1 WT, ΔVpu, Vpu LILI, Vpu ELV or Vpu 2/6A mutant together with increasing concentrations of pCR3.1 tetherin-HA expression plasmid. Cell lysates and sucrose purified viral supernatants from 50 ng tetherin input were subjected to SDS-PAGE and analyzed by Western blotting for HSP90, HIV-1 p24CA and Vpu, and analyzed by LiCor quantitative imager. Asterisk: non-specific band. (C) Infectivity of viral supernatants was assayed on HeLa-TZMbl reporter cells. Infectious virus release was plotted as β-galactosidase activity in relative light units (RLU). Error bars represent the standard deviation of three independent experiments. (D) HeLa-TZMbl cells were co-transfected with pCR3.1 Vpu-HA or indicated mutant and a GFP expression vector. Cell-surface tetherin levels were analysed 48 hours post transfection by flow cytometry. GFP positive cells were gated and tetherin levels (solid lines) were compared to un-transfected cells or transfected with indicated Vpu (dotted lines). Numbers indicate median fluorescence intensities of endogenous tetherin surface levels. The solid peak in the upper histogram in the middle of the panel represents binding of the isotype control. (E) 293T tetherin expressing cells were transfected twice over a 48 hour period with siRNA oligonucleotides directed against UBAP1 or non-targeting control. The cells were then infected with HIV-1 WT, HIV-1 Vpu LILI, HIV-1 Vpu A14L W22A or HIV-1 ΔVpu at an MOI of 2. 48 hours later, cells were lysed and immunoprecipitated with anti-tetherin antibody. Total cell lysates and precipitates were subjected to SDS-PAGE and analyzed by Western blotting for tetherin, UBAP1 and Vpu, and analyzed by ImageQuant. (TIF) [file ppat.1005141.s002.tif]

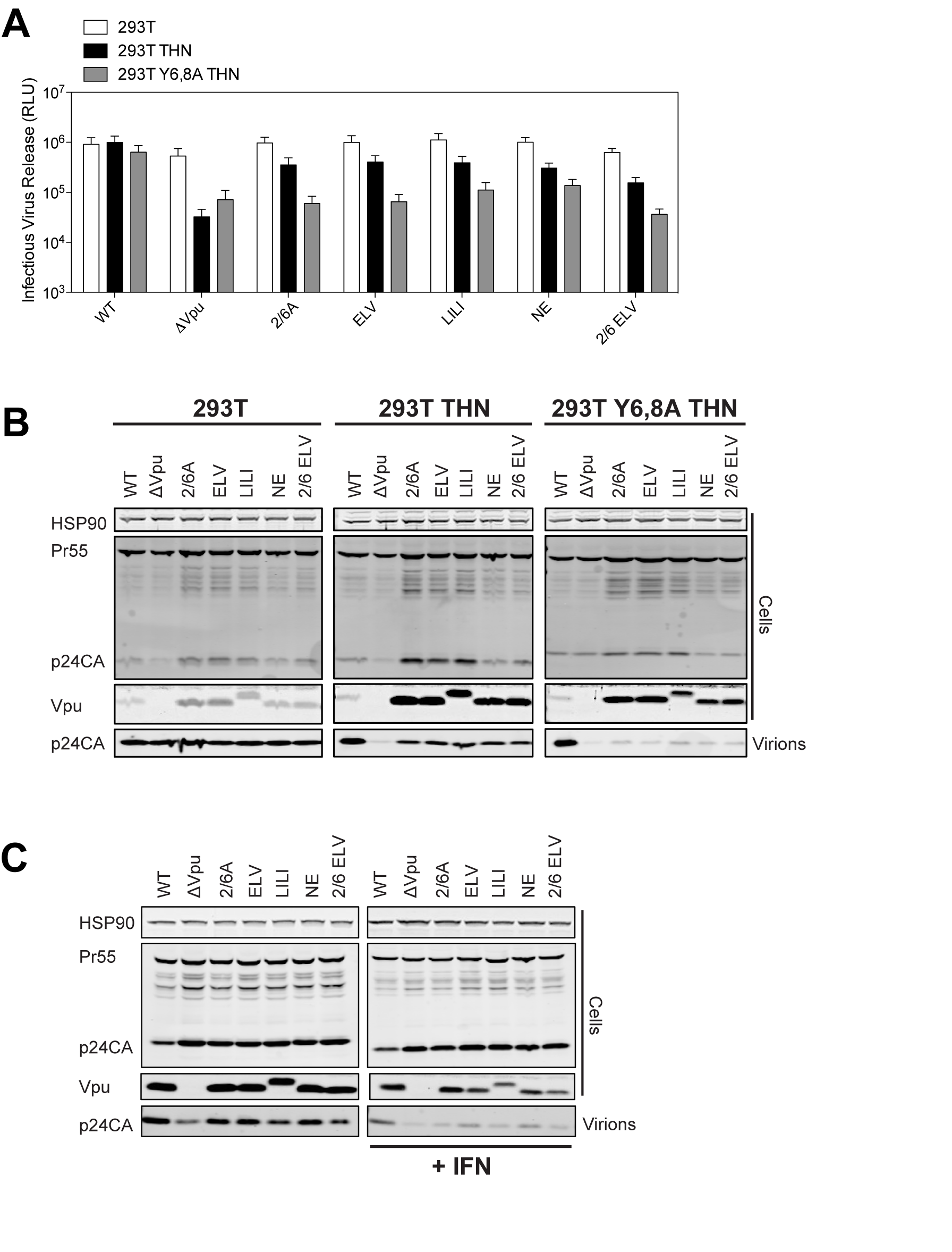

Supplement: S3 Fig — (A-B) 293T, 293T tetherin or Y6,8A tetherin cells were infected with VSV-G pseudotyped NL4.3 wt or mutant virus at an MOI of 0.8. (A) 48 hours post infection viral supernatants were assayed for infectivity using HeLa-TZMbl reporter cells as in Fig 1. Error bars represent the standard deviation of three independent experiments. (B) Cell lysates and sucrose purified viral supernatants were subjected to SDS-PAGE and analyzed by Western blotting as in Fig 1. (C) Primary human CD4+ T cells were infected with the indicated HIV-1 mutant at an MOI of 0.8. 16 h later the cells were treated or not with 5000 U/ml universal type-I interferon. Cell lysates and viral supernatants were harvested a further 24 h later and analyzed for infectivity on HeLa-TZMbl cells (A) or physical particle yield and cellular viral expression by quantitative Western blotting. (TIF) [file ppat.1005141.s003.tif]

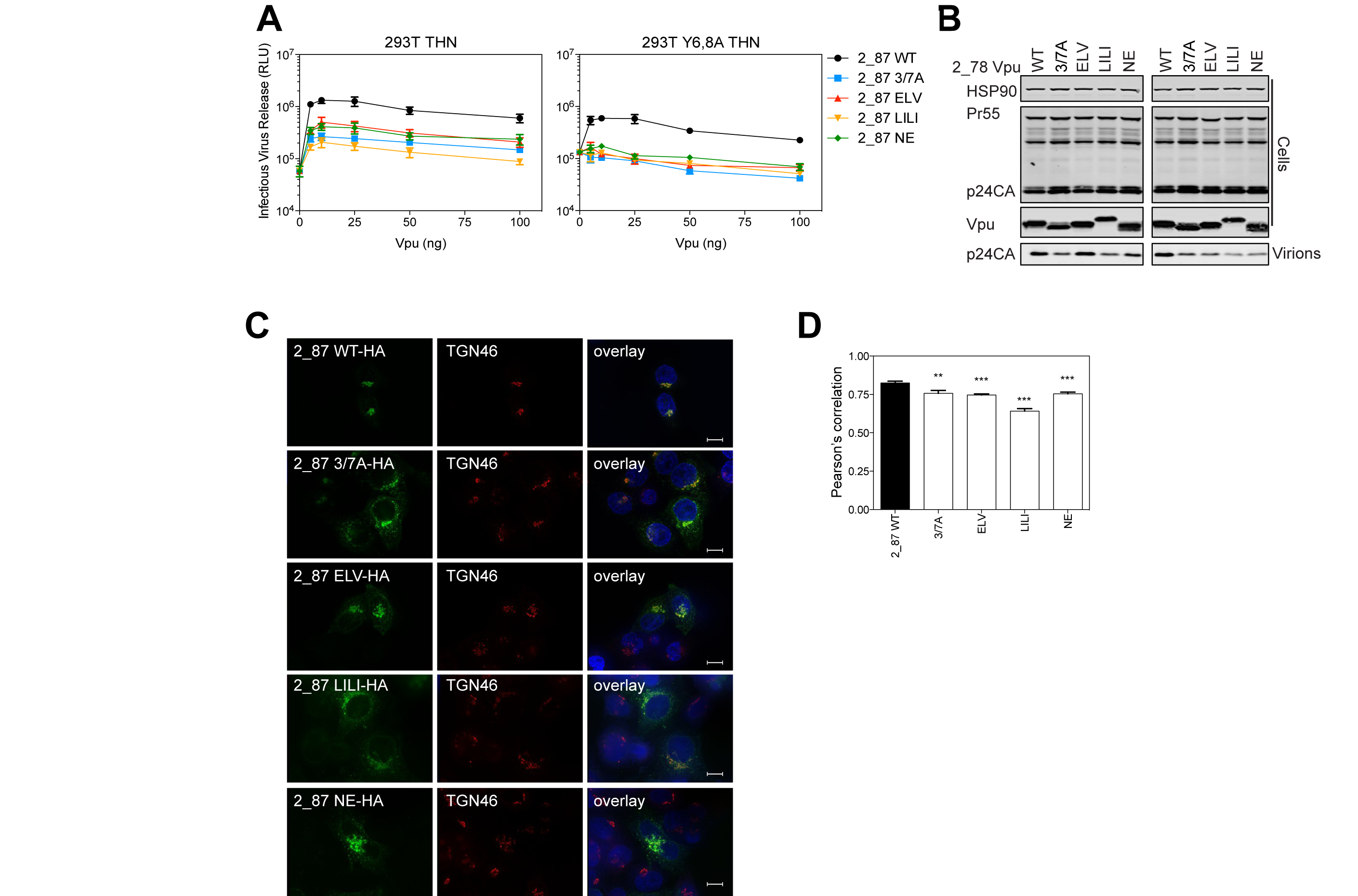

Supplement: S4 Fig — (A) 293T tetherin cells were transfected with NL4.3 ΔVpu proviral plasmid in combination with YFP expression vector and pCR3.1 2_87 Vpu or mutants thereof. 48 hours post transfection infectivity of viral supernatants was determined on HeLa-TZMbl cells as in Fig 1. (B) Cell lysates and pelleted supernatant virions from (A) were harvested and subjected to SDS-PAGE and analyzed by Western blotting for HIV-1 p24CA, Vpu and HSP90, and analyzed by LiCor quantitative imager. (C) Hela cells were transfected with 100 ng of pCR3.1 2_87 Vpu-HA or indicated mutants. 16 hours post transfection cells were fixed and stained for HA (green) and the TGN marker TGN46 (red) and examined by widefield fluorescent microscopy. Panels are of representative examples. Bars = 10 μm. (D) Z stacks were taken of all cells (n = 15), images were deconvolved using the AutoQuant X3 software and Pearson’s correlations were calculated for all Z stacks using ImageJ. Results were analyzed by unpaired 2-tailed t-test—*** P = 10–5 or lower. (TIF) [file ppat.1005141.s004.tif]

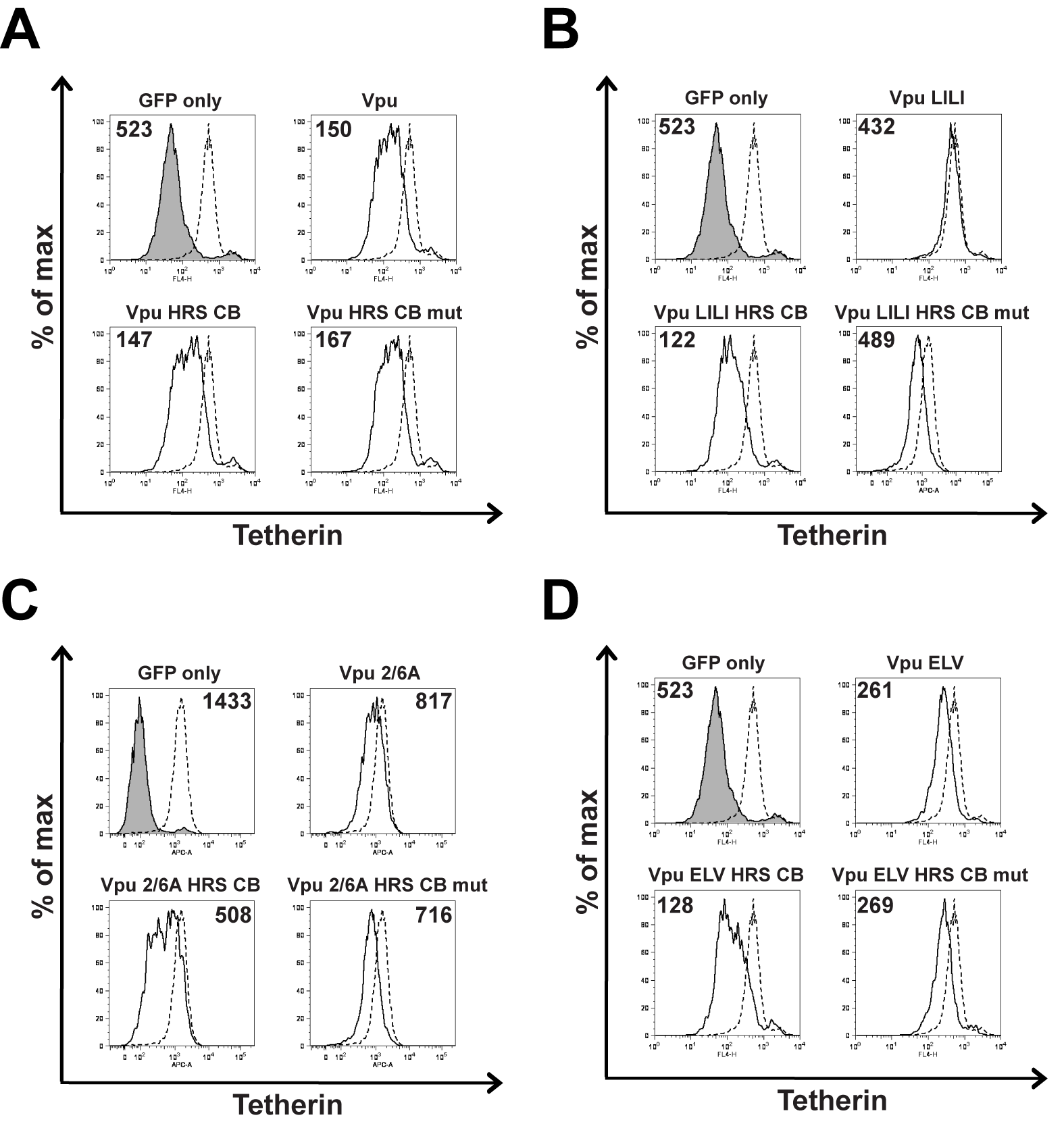

Supplement: S5 Fig — (A to D) HeLa-TZMbl cells were co-transfected with pCR3.1 Vpu-HA or indicated mutant and a GFP expression vector. Cell-surface tetherin levels were analyzed 48 hours post transfection by flow cytometry. GFP positive cells were gated and tetherin levels (solid lines) were compared to un-transfected cells or transfected with indicated Vpu (dotted lines). Numbers indicate median fluorescence intensities of endogenous tetherin surface levels. The solid peak in the upper histogram in the middle of the panel represents binding of the isotype control. (TIF) [file ppat.1005141.s005.tif]

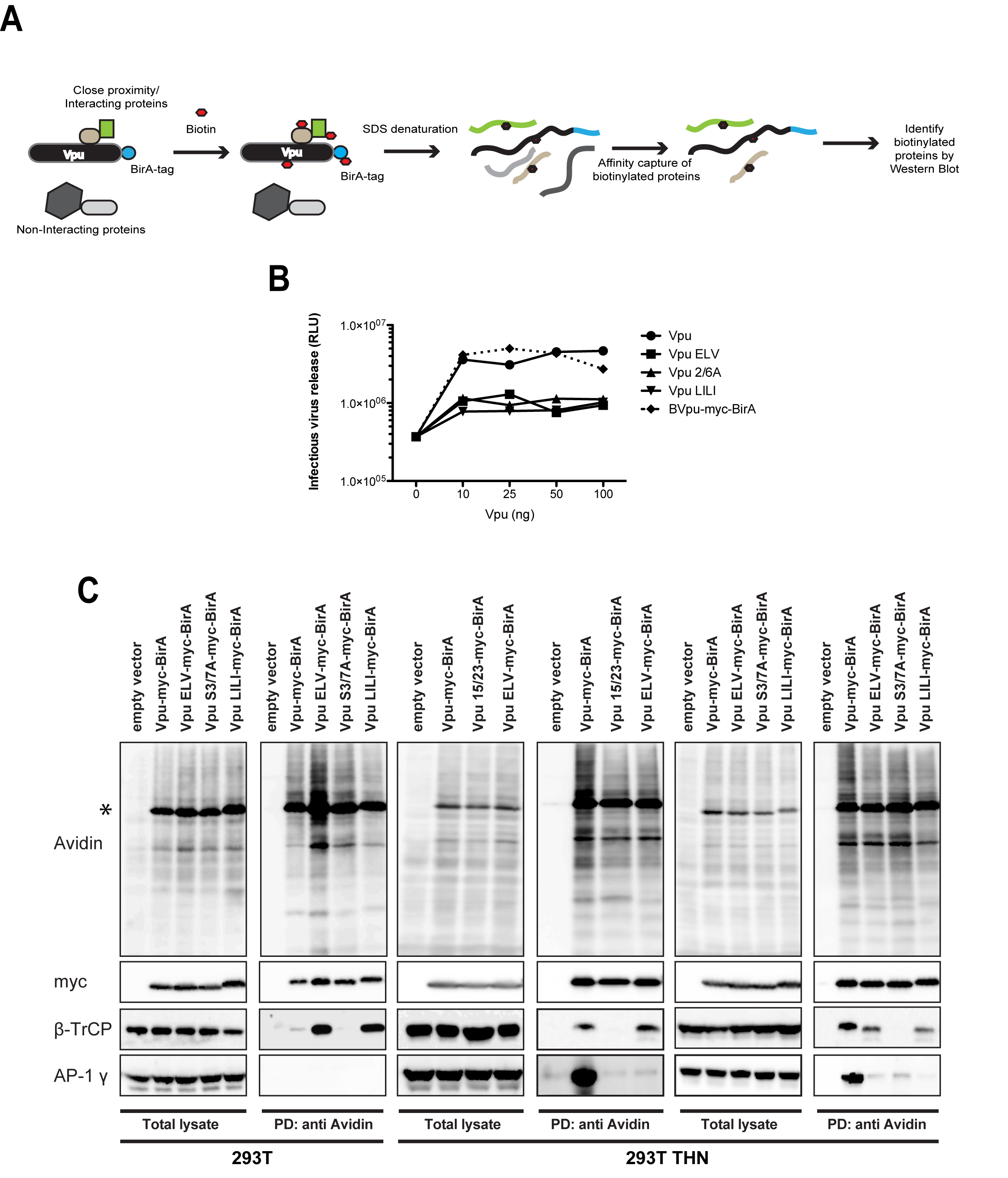

Supplement: S6 Fig — (A) Schematic representation of proximity-based biotin ligase assay. (B) 293T tetherin cells were transfected with NL4.3 ΔVpu proviral plasmid in combination with YFP expression vector and pCR3.1 Vpu or indicated mutant thereof. 48 hours post transfection infectivity of viral supernatants was determined on HeLa-TZMbl cells as in Fig 1. (C) 293T or 293T tetherin cells were transfected with Vpu-myc-BirA, B Vpu ELV-myc-BirA, B Vpu A15L/W23A-myc-BirA, B Vpu S3/7A- myc-BirA (phospho-mutant), B Vpu LILI-myc-BirA or empty vector control. 6 hours post transfection, cells were treated with 100 nM concanamycin A in the presence of 150 μM free biotin. 16 hours later, cells were washed, lysed, sonicated and biotinylated proteins were recovered on streptavidin-conjugated beads and analysed by Western blot for avidin, Vpu-myc-BirA, β-TrCP or AP-1 γ. Asterisk: Vpu-myc-BirA band. (TIF) [file ppat.1005141.s006.tif]

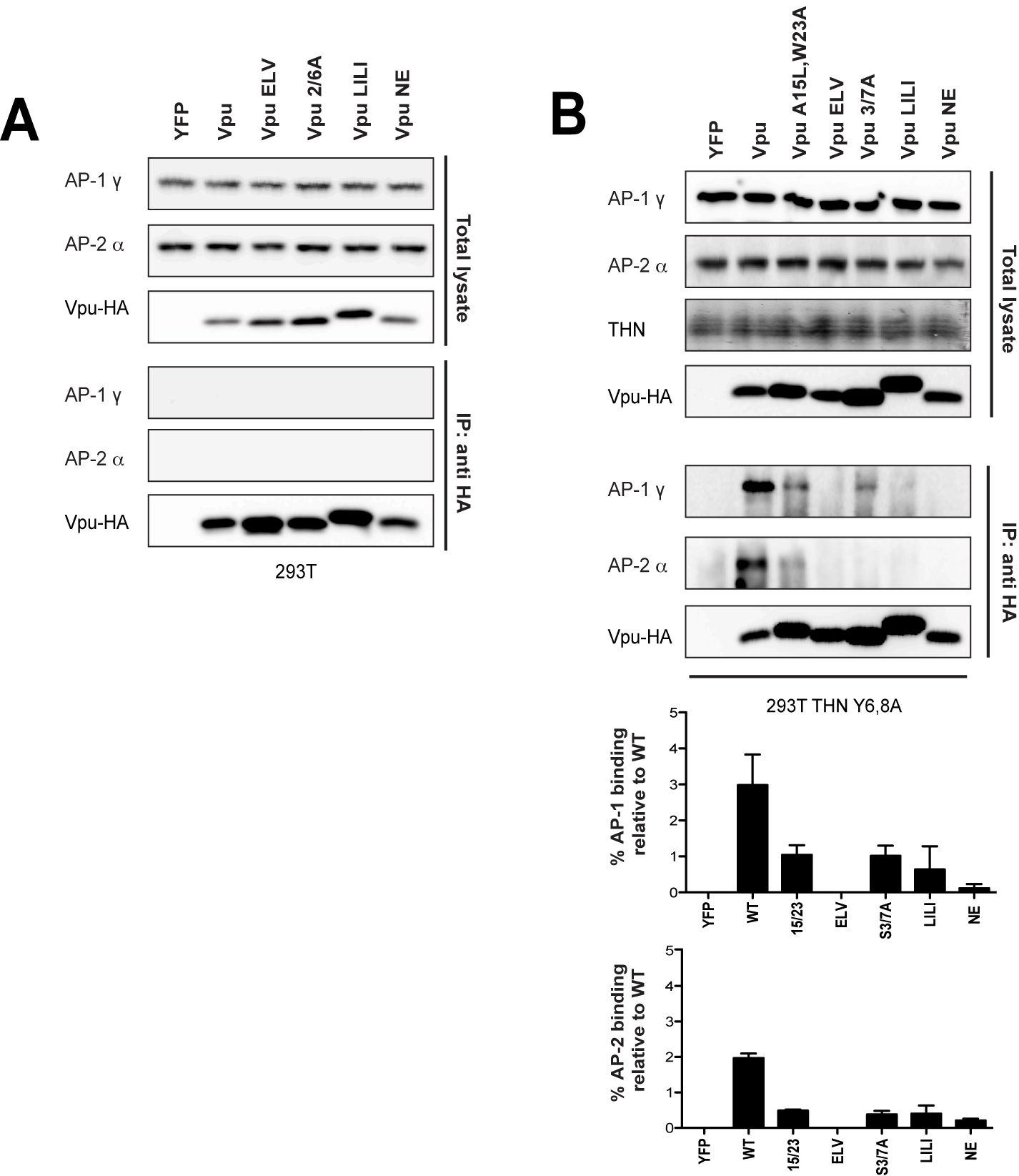

Supplement: S7 Fig — (A) 293T cells were transfected with pCR3.1 Vpu-HA, Vpu ELV-HA, Vpu 3/7A-HA, Vpu LILI-HA or Vpu NE-HA mutants. (B) 293T tetherin Y6,8A were transfected with pCR3.1 Vpu-HA, Vpu A15L/W23A-HA, Vpu ELV-HA, Vpu 3/7A-HA, Vpu LILI-HA or Vpu NE-HA mutants. 48 h post transfection, cells were lysed and cross-linked using PFA (0.05% w/v) and immunoprecipitated with anti-HA antibody. Total cell lysates and precipitates were subjected to SDS-PAGE and analyzed by Western blotting for Vpu-HA, AP-1 Ɣ or AP-2 α. Panels are of representative experiments. Histograms represent quantification of the relative AP-1 or AP-2 binding normalized to input control. Error bars represent the standard deviation of three independent experiments. (TIF) [file ppat.1005141.s007.tif]
